# Virtual Screening of Traditional Chinese Medicine Natural Product Inhibitors Targeting AQP1 for Bladder Cancer

**DOI:** 10.1002/cnr2.70570

**Published:** 2026-05-08

**Authors:** Di Liu, Qing‐Yu Zhang

**Affiliations:** ^1^ Department of Urinary Surgery Qiandongnan Prefecture People's Hospital, Affiliated Hospital of Guizhou Medical University Kaili Guizhou Province China; ^2^ Department of Urinary Surgery People's Hospital of Jinping County Jinping Guizhou Province China

**Keywords:** AQP1, bladder cancer, molecular docking, molecular dynamics, natural products

## Abstract

**Background:**

Bladder cancer (BCa) is the most common and representative type of adult urinary tract urothelial cancer, characterized by high incidence and mortality rates. It has become a major disease threatening public health, highlighting the urgent need for the development of comprehensive treatment strategies, including traditional Chinese medicine, immunotherapy, and genetic diagnostics. Aquaporin‐1 (AQP1), a water channel protein, mediates water transport across the cell membrane. Recent studies have shown that aquaporins are involved in the development and progression of malignant tumors. In particular, AQP1 plays a significant role in the pathogenesis of bladder cancer and may serve as a potential target for novel drug development. Natural products, owing to their structural diversity, represent a valuable reservoir of lead compounds for drug discovery.

**Methods and Results:**

In this study, we employed molecular docking and molecular dynamics simulations to screen a library of 2000 traditional Chinese medicine natural compounds for potential activity against the target protein AQP1. The final analysis identified 17 compounds with high binding affinity for the active site of AQP1, indicating their potential as candidate inhibitors. Among these, compounds 8 (Clematignoside), 10 (Ginsenoside Rb2), and 15 (Tannic acid) demonstrated particularly strong binding affinity and complex stability in molecular dynamics simulations.

**Conclusion:**

These findings provide a valuable foundation for the rational design of AQP1‐targeted pharmacophores and suggest promising candidates for the future development of clinical drugs for the treatment of bladder cancer. The top‐ranked compounds, especially compounds 10 and 15, warrant further experimental investigation.

## 1 Introduction

Bladder cancer (BCa) is the most common malignancy in urological surgery and is associated with a poor prognosis [1]. BCa is generally classified into muscle‐invasive and non‐muscle‐invasive types [2]. According to relevant reports, the five‐year survival rate for non‐muscle‐invasive bladder cancer is approximately 50%, whereas that for muscle‐invasive bladder cancer is even lower, with high‐risk patients having a 5‐year survival rate of only 25%–35% [3]. Although significant progress has been made in early screening and detection in recent years, the prognosis remains unfavorable due to the rapid metastasis of tumors to other organs. The initial treatment for BCa typically involves postoperative chemotherapy, with the standard regimen consisting of gemcitabine (GEM) combined with cisplatin. However, patients often develop resistance to this chemotherapy regimen over time [4]. As the average survival time for patients receiving chemotherapy is only 14 months [5], there is an urgent need for more effective therapeutic drugs to improve treatment outcomes in BCa.

Aquaporins (AQPs) are a family of transmembrane transport proteins responsible for water permeability, characterized by high selectivity and efficiency. These proteins have a molecular weight of approximately 30 kDa and comprise 13 known isoforms, designated AQP0 through AQP12. AQPs play a crucial role in osmoregulation and the maintenance of systemic water balance [6]. They are also involved in various biological processes, including epithelial fluid transport, cell migration, and cerebral edema [7]. In recent years, accumulating evidence has indicated that AQPs may serve as potential therapeutic targets. Rapid transmembrane water transport, facilitated by AQPs, is essential for tumor cell metastasis [8]. In vivo water homeostasis is tightly regulated, and disturbances in water balance have profound effects on tumor initiation and progression [9].

Aquaporin‐1 (AQP1) is a glycoprotein located on the cell membrane, primarily expressed in endothelial cells of submucosal microvessels and arterioles in the bladder [10]. It regulates the permeability of epithelial and endothelial barriers by facilitating water transport across cell membranes [11]. AQP1 is expressed in the capillary endothelium of all normal tissues, with relatively higher expression in microvascular structures. This suggests that AQP1 overexpression may be a consequence of angiogenesis and may play a critical role in the formation and clearance of tumor‐associated edema [12]. Mechanistically, AQP1 is primarily involved in regulating tumor vascular permeability, promoting angiogenesis and cell proliferation, and facilitating tumor cell migration and invasion. Aberrant expression of AQP1 has been closely linked to the occurrence and recurrence of bladder cancer [13], and it plays a key role in the progression of superficial bladder cancer [14]. Recent studies have shown that Polyporus umbellatus can inhibit bladder cancer development by modulating AQP1 expression in bladder tissues [15], while Ganoderma lucidum polysaccharides suppress tumor growth in T24 bladder cancer‐bearing mice by regulating the expression of AQP1 and AQP3 [16]. Therefore, AQP1 represents a promising therapeutic target for the treatment of bladder urothelial carcinoma. Currently, no AQP1‐targeted drugs have been approved for clinical use, underscoring the need for new therapeutic agents and strategies.

In the era of big data, the data generated during drug discovery and shared through public databases hold significant value [17]. Molecular docking is a key tool in computer‐aided drug design, used to analyze the binding patterns of ligands and receptors and predict binding affinity to screen potential lead compounds for specific targets [18]. In recent times, there is a spike in computational and structure‐based drug design studies targeting cancer therapeutics [19]. While AQP1 has been previously investigated as a potential target, most reported inhibitors are synthetic compounds or repurposed drugs. In contrast, natural products offer unique structural scaffolds and multi‐target potential that may enable novel binding modes and therapeutic strategies [20]. While molecular docking of natural product libraries is an established approach, its application to AQP1 for bladder cancer remains relatively unexplored, offering a unique avenue for discovery.

In this study, we targeted AQP1 for active site analysis and screened a library of 2000 natural products to identify high‐affinity compounds. The potential lead compounds were then evaluated by molecular dynamics simulation to assess complex stability. Compared with existing studies on AQP1 inhibitors, this study focuses on a traditional Chinese medicine‐derived natural product library, which provides greater structural diversity and potential multi‐target characteristics. Moreover, the integration of molecular docking, molecular dynamics simulations, and in silico ADMET prediction offers a comprehensive computational framework for the identification of novel AQP1 inhibitors. This study aimed to identify a novel natural product–derived compound library targeting AQP1, featuring unique structural motifs compared with existing synthetic inhibitors, thereby providing a valuable foundation for the development of new therapeutic agents against bladder cancer.

## 2 Methods

### 2.1 Receptor Preparation

**FIGURE 1:**
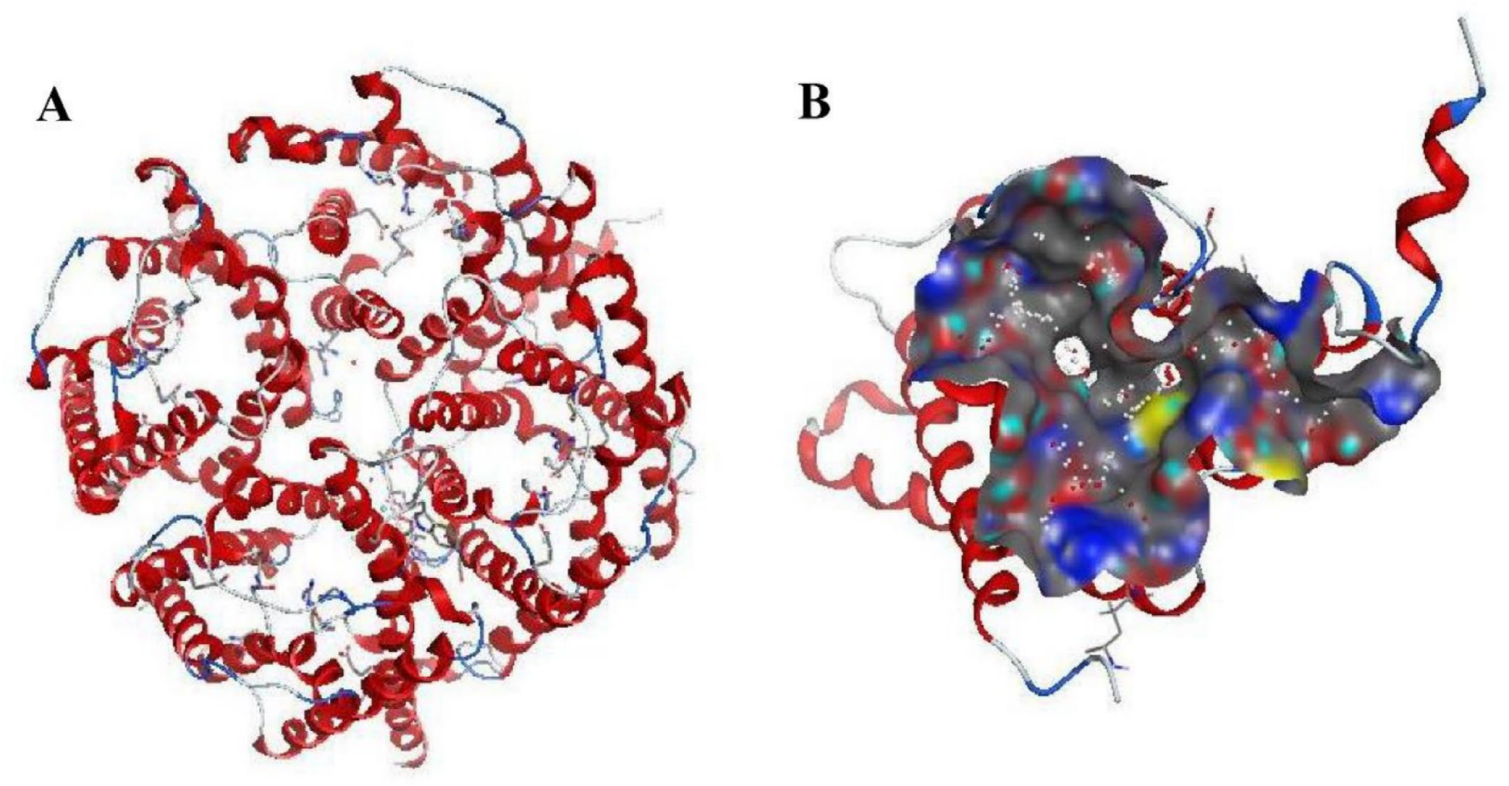
3D structure of AQP1 and active sites predicted by MOE software. (A) The overall 3D structure of the AQP1 homotetramer. (B) A close‐up view of the predicted active site in subunit A.

The 3D structure of the AQP1 target protein was downloaded from the PDB database (http://www.rcsb.org/). The PDB ID of the crystal structure with the higher resolution was 4NEF (Figure 1A). This structure represents the highest‐resolution (2.0 Å) wild‐type human AQP1 crystal structure available and is free of mutations. The selected structure does not contain mutations affecting the active site region used in this study; therefore, no additional homology modeling was required. The removal of water molecules, protonation, hydrogenation, and energy minimization of the AQP1 protein were successively performed in MOE (Molecular Operating Environment, version 2019.01). The pretreated target protein was stored in PDB format in preparation for molecular docking.

### 2.2 Ligand Preparation

We selected a natural product library consisting of 2000 compounds constructed by our laboratory based on published literature and available chemical databases. This library is currently not publicly accessible. The library was imported into MOE, where each compound underwent processing to correct errors, protonation, and 3D structure generation. Finally, the processed small molecules were saved in the MDB format in preparation for molecular docking.

### 2.3 Active Site Prediction

AQP1 is composed of four identical subunits, and the first subunit was selected as the research object. There was no co‐crystallized compound in the crystal structure of the target protein; therefore, we selected the binding site using the predictive method. The “Site Finder” option of the MOE software was used to predict active sites. We clicked “site finder” to find the binding site, and then clicked “dummies” to establish the virtual atom. The largest predicted pocket was selected as the active site for docking.

### 2.4 Molecular Docking

Molecular docking was performed in two stages. First, semi‐flexible docking was carried out using the “DOCK” module in MOE. The processed receptor and ligand files were selected. The docking parameters were set as follows: the force field was set to MMFF94x, the placement method was Triangle Matcher, and the scoring function was London dG. The top poses were retained. A lower S‐score indicates more favorable binding energy. Compounds with an *S*‐score < −8 kcal/mol were selected for further docking. In the second stage, these selected compounds were docked using the “LibDOCK” method in Discovery Studio (DS, version 2021). The same active site predicted by MOE was used, defined by a sphere with coordinates centered on the predicted site and a radius of 10 Å. The docking protocol was set to “High Quality.” The scoring function for molecular docking, LibDockScore, is a comprehensive score based on polar and nonpolar interactions; larger values indicate more stable conformations of the docking‐generated complexes and stronger affinity between the ligand and the receptor.

### 2.5 Interaction Result Analysis

Based on the lowest *S*‐score, we chose to analyze the binding modes of the top‐ranked compounds, including Compounds 8 and 15, within the AQP1 pocket. Ligand‐protein interactions were obtained by selecting “Ligand interaction” in the MOE window. The interactions of Compounds 8 and 15 were analyzed in MOE using 3D and 2D maps.

### 2.6 Molecular Dynamics Simulation

Molecular dynamics (MD) simulations of the docked complexes were performed using the DS software. The CHARMM force field was applied to each protein‐ligand complex. The CHARMM force field was selected because it is one of the most widely used and well‐validated force fields for protein‐ligand simulations, particularly in the Discovery Studio environment. And it provides a balanced treatment of both protein and small molecule interactions, particularly for membrane‐associated proteins such as AQP1. The system was solvated in an explicit TIP3P water model in a cubic periodic boundary box, with a minimum distance of 10 Å from the protein to the box edge. Counterions were added to neutralize the system. Prior to molecular dynamics simulation, energy minimization was performed to eliminate unfavorable contacts and optimize the system geometry. The MD simulation protocol consisted of three steps: system heating to 310 K over 50 ps, system equilibration for 100 ps, and a production phase of 100 ns under NPT conditions. Standard Dynamics Cascade settings were used as the default parameters, and the RMSD and RMSF values were analyzed to evaluate complex stability and residue flexibility after the simulation, and the results were visualized using graphical plots.

### 2.7 In Silico ADMET and Drug‐Likeness Prediction

To evaluate the potential drug‐likeness and pharmacokinetic profiles of the screened compounds, in silico Absorption, Distribution, Metabolism, Excretion, and Toxicity (ADMET) predictions were conducted. Key pharmacokinetic parameters, including oral bioavailability (OB), Caco‐2 permeability, blood–brain barrier (BBB) penetration, drug‐likeness (DL), cytochrome P450 2D6 (CYP2D6) inhibition, and hepatotoxicity, were retrieved for the top candidate compounds from the Traditional Chinese Medicine Systems Pharmacology Database and Analysis Platform (TCMSP, https://tcmsp‐e.com/). TCMSP is a widely recognized repository that provides systematically predicted ADMET properties for natural products, facilitating the early‐stage evaluation of their therapeutic potential. Compounds with an OB ≥ 30% were considered to have high oral bioavailability, while those with OB between 10% and 30% were considered moderate. Lipinski's Rule of Five (molecular weight ≤ 500, ALogP ≤ 5, number of hydrogen bond donors ≤ 5, number of hydrogen bond acceptors ≤ 10) was applied to assess drug‐likeness for oral administration, using the molecular properties provided by TCMSP.

## 3 Results

### 3.1 Active Site Prediction

After the protein structure was prepared, the active site was predicted using the MOE software. The largest predicted pocket was selected as the active site for docking. The predicted active site of the target protein consists of amino acids including GLU3 ALA8 PHE9 ALA12 VAL56 GLN57 LEU59 GLY60 HIS61 ILE62 SER63 GLY64 ALA65 HIS66 VAL71 CYS75 VAL77 GLY78 CYS79 HIS80 VAL81 SER82 ARG85 TYR89 ILE145 PHE146 ALA147 SER148 TH R149 ASP150 GLU151 ARG152 ARG153 GLY154 GLU155 ASN156 PRO157 GLY158 THR159 PRO160 ALA161 ILE164 TYR219 ASN220 TYR221 PHE224 PRO225 PRO226 LYS228 (Figure 1B).

### 3.2 Molecular Docking Results

**FIGURE 2:**
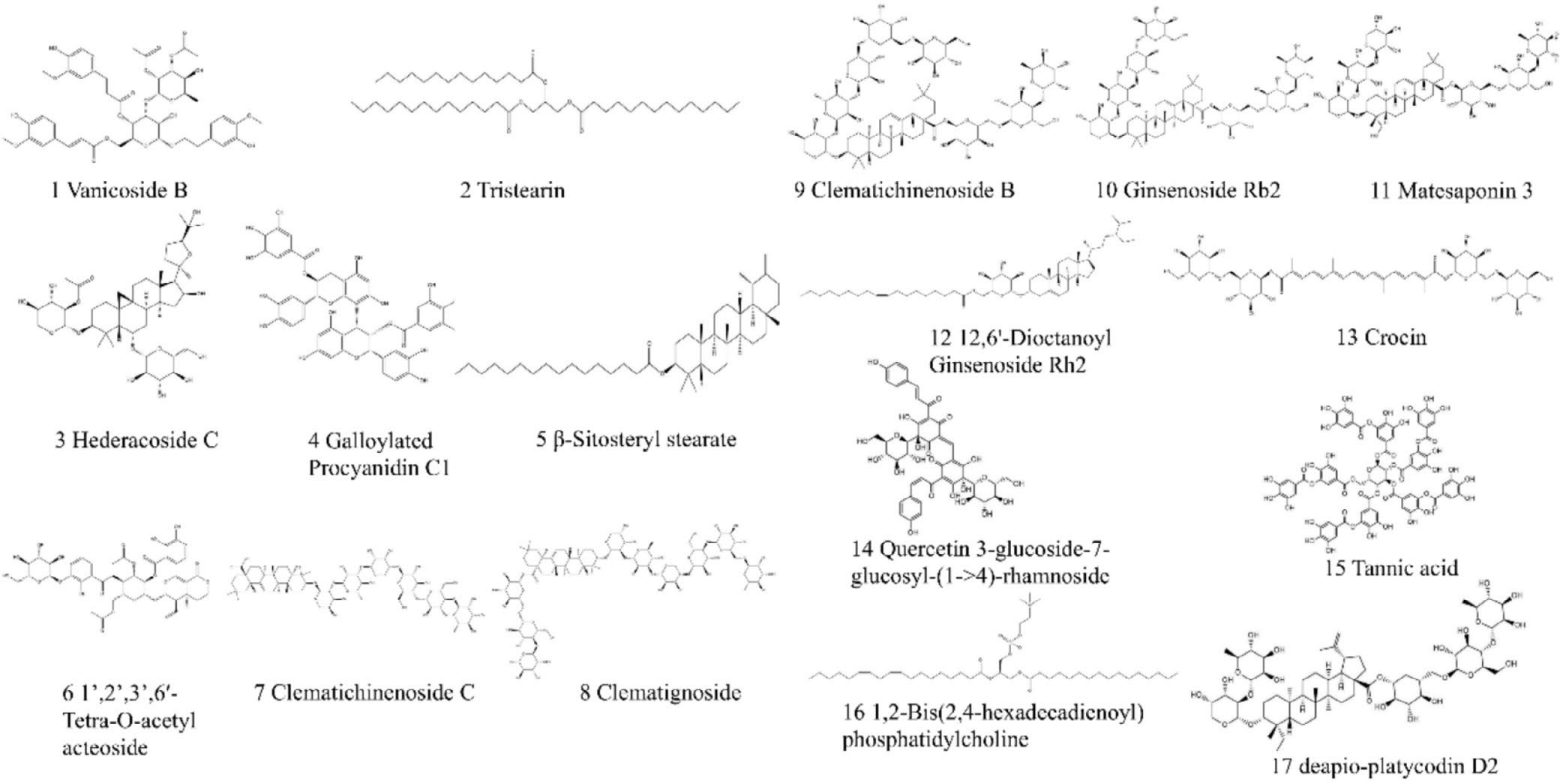
Chemical structures of the 17 potentially active natural compounds identified as high‐affinity binders for AQP1 through molecular docking screening.

The prepared ligand library was docked against the predicted AQP1 active site using MOE software. A lower value of S in the docking result indicates that the ligand is more strongly bound to the receptor and can be used as a potential active substance. The S‐score of less than −8 was used as the screening criterion, resulting in a total of 17 compounds, listed in Table 1 and Figure 2. Then, the number of conformations was set using the LibDock tool through “High Quality” docking. The docking result was measured by the “LibDockScore” for the docking of different conformations with the protein receptor. Similarly, DS software docking results are presented in Table 1. We listed the compounds with a LibDOCK Score greater than 100 as ligands with strong affinity. The results showed excellent affinity except for Compound 7.

**TABLE 1:** Molecular docking scores and molecular dynamics simulation results for the 17 natural compounds with high affinity for AQP1.

No.	Compound names	S (kcal/mol)	LibDOCK score	Average RMSD*
1	Vanicoside B	−8.9	167.2	1.46
2	Tristearin	−8.4	153.6	0.94
3	Hederacoside C	−8	119.6	0.93
4	Galloylated Procyanidin C1	−8.3	163.7	0.91
5	β‐Sitosteryl stearate	−8.4	147.7	0.92
6	1′,2′,3′,6′‐Tetra‐O‐acetyl acteoside	−8.6	160.3	0.99
7	ClematichinenosideC	−8.3	162.1	1.44
8	Clematignoside	−9.9	193.4	1.44
9	Clematichinenoside B	−8	194.7	0.95
10	Ginsenoside Rb2	−8	125.6	0.84
11	Matesaponin 3	−9	158.1	0.94
12	12,6′‐Dioctanoyl Ginsenoside Rh2	−8.2	190.6	0.93
13	Crocin	−9	100.6	0.95
14	Quercetin 3‐glucoside‐7‐glucosyl‐(1→4)‐rhamnoside	−8.2	140.6	0.98
15	Tannic acid	−9.2	139.3	0.89
16	1,2‐Bis‐(2,4‐hexadecadienoyl) phosphatidylcholine	−8.8	133.6	1.0
17	Deapio‐platycodin D2	−8.4	170.2	1.1

* Average RMSD (Å): The average root‐mean‐square deviation of the ligand‐receptor complex during the 100 ns molecular dynamics simulation. Lower values suggest greater complex stability.

### 3.3 Protein‐Ligand Interaction

**FIGURE 3:**
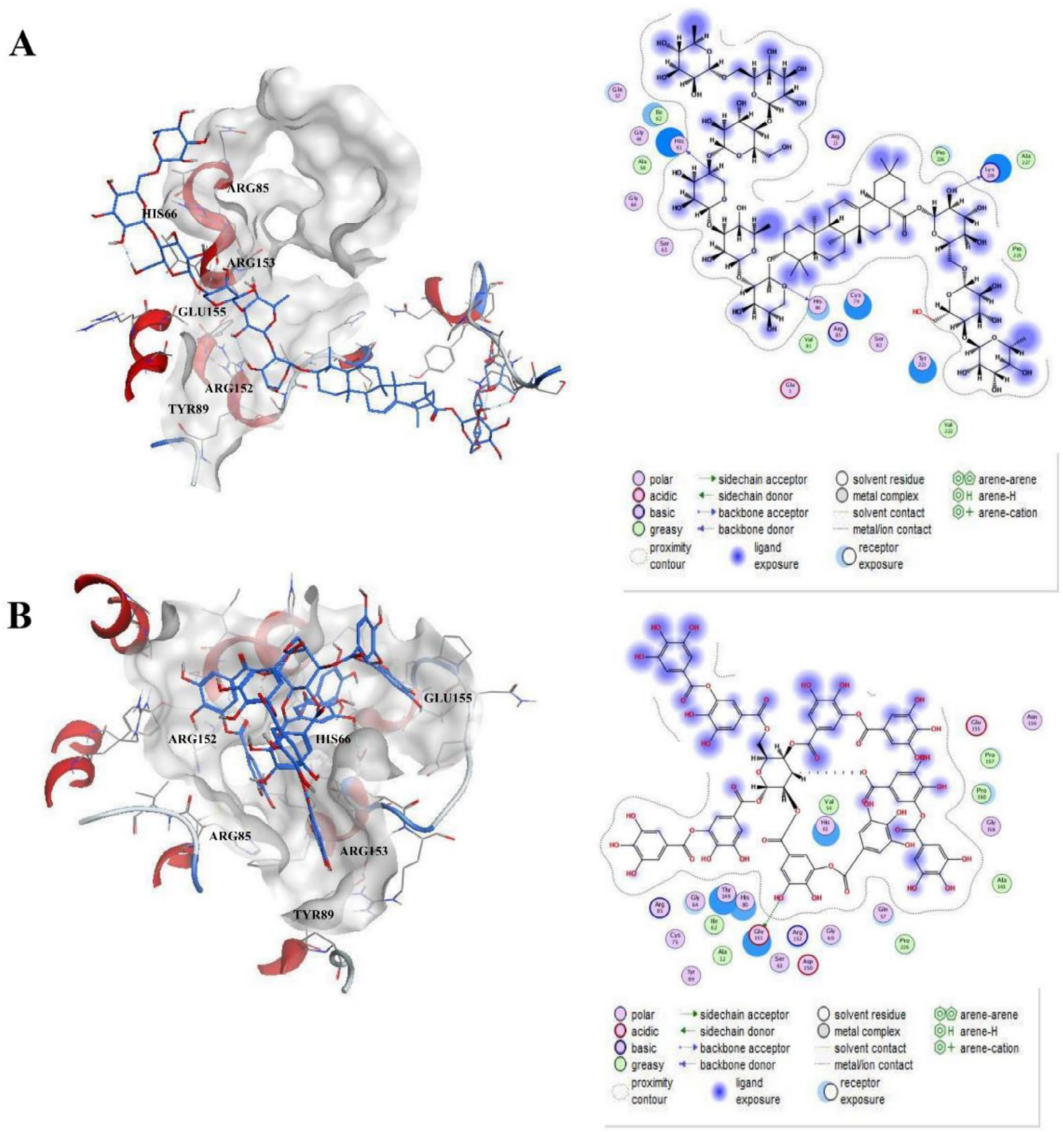
2D and 3D maps of top compounds with the AQP1 binding pocket in MOE software. (A) 2D and 3D interaction diagrams of Compound 8 (Clematignoside) with AQP1. Hydrogen bonds are shown as red arrows, and hydrophobic interactions are shown as blue arcs. (B) 2D and 3D interaction diagrams of Compound 15 (Tannic Acid) with AQP1. Key interacting residues (HIS66, ARG85, TYR89, ARG152, ARG153, and GLU155) are highlighted.

To understand the binding mechanisms, we analyzed the interactions of the top‐ranking compounds with the AQP1 active site. Molecular docking was performed in different conformations by analyzing the non‐bonded interactions of the ligands in the active pocket. The MOE software analyzes the binding patterns of proteins and compounds, and simulates the specific spatial locations and interaction relationships of compounds and proteins as they bind. Here, we analyzed the interactions of Compounds 8 (Clematignoside) and 15 (Tannic Acid) with active site residues using MOE software. Compound 8 is a triterpenoid saponin from the roots of Clematis manshurica in China, which is a commonly used herb in Asia and has anti‐inflammatory and antioxidant effects [21, 22]. Recent studies have reported that Compound 15 has significant anti‐angiogenic activity in non‐small cell lung cancer (NSCLC) [23]. The MOE docking scores for Compound 8 and Compound 15 were −9.9 kcal/mol and −9.2 kcal/mol, respectively. From the docking results, the two compounds interact with multiple amino acid residues in the pocket, with Compound 8 interacting with more residues than Compound 15 (Figure 3A,B).

Compounds 8 and 15 were analyzed in detail due to high scores and stability. Compound 8 (MOE score: −9.9 kcal/mol) and Compound 15 (MOE score: −9.2 kcal/mol) formed extensive interactions (hydrogen bonds, hydrophobic contacts) with key residues including HIS66, ARG85, TYR89, ARG152, ARG153, and GLU155 (Figure 3A,B).

### 3.4 Molecular Dynamics Simulation Results

No single molecular docking strategy in the field of molecular docking has an absolute advantage. Molecular docking removes solvent molecules and therefore does not mimic the actual binding environment of ligand‐receptor interactions. To evaluate the stability of the ligand‐receptor complexes in a solvated, dynamic environment, we performed 100 ns MD simulations. The root mean square deviation (RMSD) of the protein backbone and the ligand relative to the initial docked pose was calculated as an indicator of system stability. Lower and more stable RMSD values suggest a more stable complex. MD studies are increasingly being employed to study protein‐ligand interactions [24]. Mean RMSD values for all ligand‐receptor complexes are also presented in Table 1. The results demonstrated that the complexes of compounds 10 (Ginsenoside Rb2) and 15 were highly stable. In contrast, although Compounds 1 and 8 had significant affinity in docking, their complexes showed higher RMSD fluctuations, suggesting weaker stability. To further evaluate the flexibility of residues in the AQP1–ligand complexes, the root mean square fluctuation (RMSF) of each amino acid in the receptor protein was calculated, and RMSF analysis was performed for the representative compounds (Compounds 8, 10, and 15). The RMSF value indicates the degree of freedom of amino acid movement during the molecular dynamics simulation. As shown in Figure S1, most residues exhibited relatively low fluctuation values (generally below 1.0 Å), indicating overall structural stability of the complexes during the simulation. Higher fluctuations were primarily observed in loop regions, which is consistent with their intrinsic flexibility. Importantly, residues within the predicted binding pocket remained relatively stable across all three complexes, supporting the reliability of the docking interactions. The RMSF values for each amino acid residue of that each complex are presented in the Table S1.

### 3.5 In Silico ADMET and Drug‐Likeness Prediction

In silico ADMET profiling was conducted for the top‐ranked compounds using the Traditional Chinese Medicine Systems Pharmacology (TCMSP) database (Table 2). The analysis revealed distinct profiles among the hits. The highest‐affinity compounds, Compounds 8 and 10, were predicted to have low oral bioavailability (OB = 7.57% and 6.02%, respectively). They exhibited three and two violations of Lipinski's Rule of Five, respectively, primarily due to their high molecular weight, high calculated lipophilicity (ALogP), and excessive numbers of hydrogen bond donors/acceptors. In contrast, compounds 2 (Tristearin) and 5 (β‐Sitosteryl stearate) demonstrated favorable predicted oral bioavailability, yet each also showed two Lipinski violations owing to similar physicochemical challenges. Compound 15, with relatively lower predicted OB, likewise presented two violations. Consequently, while these molecules are not suitable as direct drug candidates in their current form, they serve as valuable pharmacophore models for future structural optimization. This critical finding underscores a key implication of our study: the identified hits function primarily as high‐affinity molecular probes and pharmacophore templates to inform future rational drug design, rather than as development‐ready candidates.

**TABLE 2:** ADMET and drug‐likeness predictions for top 5 compounds (Based on TCMSP data & docking results).

Compound	OB (%)	Caco‐2	BBB	DL	CYP2D6 inhibitor	Hepatotoxicity	Lipinski‘s rule violations
8	7.57	−8.20	−10.2	0.11	No	Low	3
10	6.02	−3.92	−5.43	0.04	No	Low	2
15	7.89	−6.10	−7.29	0.03	No	Low	3
2	15.13	0.54	−1.12	0.33	No	Low	2
5	40.39	1.39	1.11	0.85	No	Low	2

## 4 Discussion

A new drug requires a lot of effort and money from research and development to market approval. Computer‐aided drug design (CADD) is an important approach to reduce cost and time in drug development, especially for small molecule and protein drugs [25, 26, 27]. Molecular docking is often used to predict the binding poses of small molecules to their targets as well as their affinity [28]. This study utilized an integrated computational approach, combining molecular docking, molecular dynamics simulations, and in silico ADMET predictions, to identify natural product inhibitors of AQP1 for bladder cancer therapy. We successfully screened a library of 2000 natural compounds and identified 17 with high binding affinity for AQP1. Among these, compounds 8 (Clematignoside), 10 (Ginsenoside Rb2), and 15 (Tannic Acid) emerged as particularly promising candidates based on their robust docking scores and stability profiles.

AQPs are a family of highly selective transmembrane proteins that primarily facilitate the transport of water across cell membranes [29]. There is growing evidence that AQPs play a critical role in cancer metastasis and progression. Upregulation of AQPs has been documented in various tumor types, including bladder, breast, prostate, lung, liver, cervical, ovarian, skin, gastric, and colorectal cancers [30]. Clinically, AQPs hold significant potential as both biomarkers and therapeutic targets. Particularly, AQP1 regulates the permeability of epithelial and endothelial barriers by facilitating water movement across the cell membrane, is increasingly recognized for its roles in cancer biology [12, 30, 31]. In bladder cancer, AQP1 overexpression is linked to tumor stage, angiogenesis, and metastasis [13, 32, 33]. Our study reinforces the therapeutic potential of AQP1 inhibition. The mechanistic role of AQP1 in bladder cancer involves enhancing vascular permeability and facilitating tumor cell migration [12, 31].

One study reported that AQP1 promotes interstitial fluid pressure and vascular hyperpermeability in bladder, pancreatic, and breast cancers [12]. In addition, AQP1 is involved in the formation of tumor‐associated exudates or edema that stimulate angiogenesis [12]. Pan demonstrated that upregulation of AQP1 exacerbates the development and progression of bladder cancer. By inhibiting AQP1, these pro‐tumorigenic processes may be disrupted. For instance, silencing AQP1 has been shown to suppress tumor growth and angiogenesis in experimental models [33], and combining AQP1 inhibition with chemotherapy such as mitomycin C could overcome resistance [34]. Similarly, Liu found that AQP1 expression reflects tumor grade and progression in bladder cancer, suggesting its potential utility as a target for specific diagnostic and therapeutic interventions [35]. Targeting AQP1 directly or indirectly may offer an effective strategy for the treatment of bladder cancer. Our findings align with this strategy, providing specific natural product candidates for achieving AQP1 inhibition. The detailed interaction analysis revealed that these compounds (Compounds 8, 10, and 15) bind to key residues in the AQP1 channel, potentially obstructing its water‐transport function. The RMSF analysis further confirmed that residues within the binding pocket remained relatively stable, supporting the robustness of the predicted binding modes.

While molecular docking successfully identified several natural products with high predicted affinity for AQP1, subsequent in silico ADMET analysis provided an essential reality check. The top hits, including Clematignoside and Ginsenoside Rb2, are triterpenoid saponins—a class valued for potent target engagement but often plagued by suboptimal pharmacokinetics due to high molecular weight and lipophilicity, resulting in drug‐likeness rule violations. This is a common challenge in natural product‐based drug discovery. Our work thus provides a clear roadmap: the conserved core structure and interaction patterns of these saponins with AQP1 (Figure 3) should be extracted to design and synthesize simplified, drug‐like analogs that retain the key binding motifs while reducing molecular weight and lipophilicity. Compounds like Tannic Acid, which also shows rule violations but may possess favorable scaffold properties, could serve as tool compounds for in vitro validation of AQP1 inhibition.

The novelty of our work lies in the systematic virtual screening of a TCM‐derived natural product library against AQP1, a target with no clinically approved drugs. Compared to known synthetic AQP1 inhibitors, the identified natural products, such as Clematignoside and Tannic Acid, offer unique structural complexity and potential for multi‐target effects, which could be advantageous for managing complex diseases like cancer. In addition, although molecular docking and MD simulations provide strong theoretical evidence, we acknowledge that the lack of experimental validation in vitro or in vivo is a limitation of the present study. Furthermore, the MD simulations were performed as single 100 ns runs for each complex due to computational resource constraints. Performing replicate simulations in future studies would further improve the robustness and reproducibility of the results. The translational relevance of our findings depends on future biological assays to confirm AQP1 inhibition and anticancer efficacy. However, the comprehensive computational workflow, including stability assessment via MD and preliminary drug‐likeness evaluation, strengthens the credibility of our hits and provides a rational prioritization for subsequent experimental work. The ADMET and Lipinski's Rule of Five predictions for the top compounds suggest that several, including the highly stable Compounds 10 and 15, possess promising drug‐like properties, supporting their potential for further development. Future work will focus on the experimental validation of these top hits in cell‐based and animal models of bladder cancer.

## 5 Conclusion

In conclusion, this study demonstrates that AQP1 is a viable target for virtual screening of natural product inhibitors. Using a combined computational approach, we identified 17 natural compounds with high binding affinity for AQP1, with Compounds 10 (Ginsenoside Rb2) and 15 (Tannic Acid) showing particularly stable interactions in molecular dynamics simulations. These compounds represent promising starting points for the development of novel AQP1‐targeted therapies for bladder cancer. While this study provides a strong computational foundation, further experimental validation is essential. Future studies will focus on experimental validation of these candidate compounds, including in vitro and in vivo assays, as well as structural optimization based on the identified pharmacophore features to improve drug‐likeness and therapeutic potential. Additionally, structure–activity relationship (SAR) studies based on the identified scaffolds, particularly those of Compound 8 (Clematignoside) and Compound 15 (Tannic acid), may guide the optimization of drug‐like properties and pharmacokinetic profiles. The combination of AQP1 inhibition with existing chemotherapy regimens also warrants investigation as a potential strategy to overcome drug resistance. The findings provide a valuable basis for future drug screening, pharmacophore modeling, and experimental investigations, ultimately contributing to the expanding arsenal of potential therapeutics against this challenging disease.

## Author Contributions

**Di Liu:** investigation, writing – original draft, data curation, funding acquisition. **Qing‐Yu Zhang:** investigation, writing – review and editing. All authors have read and approved the final version of this manuscript.

## Funding

This work was supported by the National Natural Science Foundation of China, NO. 82271835; Institutional research project of Qiandongnan Prefecture People's Hospital, NO. 202301.

## Conflicts of Interest

The authors declare no conflicts of interest.

## Supporting information


**Figure S1:** RMSF plots of AQP1 residues in complex with Compounds 8, 10, and 15 during 100 ns molecular dynamics simulations. The x‐axis represents residue indices, and the y‐axis represents RMSF values (Å).
**Table S1:** The RMSF raw values for each amino acid residue of each complex.

## Data Availability

The data that support the findings of this study are available from the corresponding author upon reasonable request.
