# Supplementary material for: Virtual Screening of Traditional Chinese Medicine Natural Product Inhibitors Targeting AQP1 for Bladder Cancer
Source: Cancer Rep (Hoboken). 2026 May 8;9(5):e70570. doi: 10.1002/cnr2.70570 (PMC13154915; doi:10.1002/cnr2.70570)
Supplement: Supplementary file 1 — Figure S1: RMSF plots of AQP1 residues in complex with Compounds 8, 10, and 15 during 100 ns molecular dynamics simulations. The x‐axis represents residue indices, and the y‐axis represents RMSF values (Å). Table S1: The RMSF raw values for each amino acid residue of each complex. [file CNR2-9-e70570-s001.docx]

**Supplementary Figure S1**. RMSF plots of AQP1 residues in complex with Compounds 8, 10, and 15 during 100 ns molecular dynamics simulations. The x-axis represents residue indices, and the y-axis represents RMSF values (Å).

**Supplementary Table S1.** The RMSF raw values for each amino acid residue of each complex

|  | RMSD (Å) | | | | | | | | | | | | | | | | |
| --- | --- | --- | --- | --- | --- | --- | --- | --- | --- | --- | --- | --- | --- | --- | --- | --- | --- |
| Residue | Compound 1 | Compound 2 | Compound 3 | Compound 4 | Compound 5 | Compound 6 | Compound 7 | Compound 8 | Compound 9 | Compound 10 | Compound 11 | Compound 12 | Compound 13 | Compound 14 | Compound 15 | Compound 16 | Compound 17 |
| ACE1 | 0.921853 | 1.02354 | 0.73667 | 0.592634 | 1.38459 | 0.990242 | 0.784358 | 1.38619 | 1.34673 | 0.913478 | 0.615735 | 0.651981 | 1.36214 | 1.3713 | 0.861933 | 0.76868 | 1.37231 |
| SER2 | 0.631582 | 0.637696 | 0.549259 | 0.608967 | 0.851567 | 0.601576 | 0.682633 | 0.938955 | 1.14751 | 0.684239 | 0.569635 | 0.558339 | 0.80843 | 0.852093 | 0.733278 | 0.55401 | 0.614547 |
| GLU3 | 0.572247 | 0.672448 | 0.624811 | 0.660036 | 0.485761 | 0.50517 | 0.50912 | 0.890587 | 0.503406 | 0.481647 | 0.693063 | 0.461317 | 0.636273 | 0.801 | 0.577541 | 0.606356 | 0.597335 |
| LEU4 | 0.518411 | 0.476163 | 0.56706 | 0.59432 | 0.42564 | 0.453871 | 0.533441 | 0.735783 | 0.426954 | 0.824189 | 0.600837 | 0.600277 | 1.03975 | 0.703585 | 0.560258 | 0.496822 | 0.855271 |
| ARG5 | 0.868045 | 0.653804 | 0.859544 | 0.929786 | 0.692753 | 0.698906 | 0.706899 | 0.714854 | 0.616444 | 0.939147 | 0.634093 | 0.614213 | 1.04508 | 1.11026 | 0.87877 | 0.58525 | 0.98475 |
| SER6 | 0.523471 | 0.428085 | 0.809352 | 0.656053 | 0.543163 | 0.506648 | 0.439779 | 0.496712 | 0.360986 | 0.526482 | 0.542466 | 0.449432 | 0.733287 | 0.483905 | 0.388554 | 0.388482 | 0.660109 |
| ILE7 | 0.357454 | 0.369275 | 0.679677 | 0.78155 | 0.729327 | 0.738061 | 0.546769 | 0.672757 | 0.533788 | 0.468015 | 0.531255 | 0.388449 | 0.796491 | 0.763038 | 0.581554 | 0.461694 | 0.614467 |
| ALA8 | 0.347841 | 0.309623 | 0.669498 | 0.636266 | 0.559194 | 0.488867 | 0.559483 | 0.578443 | 0.290986 | 0.406439 | 0.572472 | 0.35693 | 0.613699 | 0.51257 | 0.374468 | 0.369869 | 0.517452 |
| PHE9 | 0.326944 | 0.379422 | 0.569502 | 0.532625 | 0.454797 | 0.452597 | 0.436518 | 0.639971 | 0.330551 | 0.402146 | 0.446735 | 0.54862 | 0.515257 | 0.402314 | 0.467917 | 0.392451 | 0.575355 |
| SER10 | 0.259557 | 0.386697 | 0.587091 | 0.521522 | 0.390651 | 0.408703 | 0.589531 | 0.491847 | 0.349486 | 0.397096 | 0.434626 | 0.477149 | 0.588009 | 0.383073 | 0.447913 | 0.384246 | 0.558769 |
| ARG11 | 0.530991 | 0.419127 | 0.415989 | 0.624737 | 0.433949 | 0.545541 | 0.518113 | 0.379751 | 0.386304 | 0.486722 | 0.576507 | 0.612975 | 0.622262 | 0.520631 | 0.582898 | 0.555192 | 0.524688 |
| ALA12 | 0.15465 | 0.309495 | 0.365552 | 0.423738 | 0.364496 | 0.422796 | 0.410111 | 0.2829 | 0.246118 | 0.293884 | 0.317344 | 0.430845 | 0.396604 | 0.331946 | 0.318148 | 0.376614 | 0.329043 |
| VAL13 | 0.330081 | 0.373198 | 0.532439 | 0.481363 | 0.39719 | 0.423109 | 0.581754 | 0.360427 | 0.326412 | 0.410677 | 0.327221 | 0.334168 | 0.514299 | 0.386011 | 0.383701 | 0.485691 | 0.371931 |
| PHE14 | 0.510116 | 0.566305 | 0.599001 | 0.623394 | 0.806806 | 0.710533 | 0.449408 | 0.363041 | 0.583781 | 0.539358 | 0.533654 | 0.555237 | 0.620214 | 0.716966 | 0.487734 | 0.691447 | 0.499207 |
| ALA15 | 0.250988 | 0.256407 | 0.320878 | 0.23447 | 0.346278 | 0.533245 | 0.324509 | 0.271182 | 0.308297 | 0.322728 | 0.457244 | 0.408535 | 0.305867 | 0.398864 | 0.405158 | 0.278176 | 0.268944 |
| GLU16 | 0.337418 | 0.306886 | 0.288063 | 0.423701 | 0.463586 | 0.389432 | 0.371511 | 0.26138 | 0.321414 | 0.260284 | 0.354369 | 0.453135 | 0.286665 | 0.344691 | 0.422639 | 0.289567 | 0.360922 |
| PHE17 | 0.484358 | 0.389425 | 0.385075 | 0.351958 | 0.430105 | 0.661054 | 0.442488 | 0.361869 | 0.431979 | 0.391322 | 0.63322 | 0.421748 | 0.295616 | 0.409763 | 0.450969 | 0.346542 | 0.390019 |
| LEU18 | 0.555343 | 0.353726 | 0.389036 | 0.423753 | 0.374159 | 0.608702 | 0.465222 | 0.416079 | 0.41854 | 0.456643 | 0.433345 | 0.53918 | 0.469628 | 0.480876 | 0.48276 | 0.447697 | 0.371626 |
| ALA19 | 0.214354 | 0.223284 | 0.275656 | 0.48376 | 0.33412 | 0.439259 | 0.316274 | 0.331398 | 0.325799 | 0.302171 | 0.295462 | 0.465855 | 0.270269 | 0.243811 | 0.320714 | 0.294167 | 0.250374 |
| THR20 | 0.327226 | 0.245783 | 0.278566 | 0.350384 | 0.28181 | 0.36467 | 0.357732 | 0.317385 | 0.301308 | 0.282279 | 0.279198 | 0.447007 | 0.220087 | 0.267396 | 0.235394 | 0.235645 | 0.279003 |
| LEU21 | 0.428875 | 0.316998 | 0.355119 | 0.366533 | 0.415235 | 0.373116 | 0.417317 | 0.358144 | 0.294913 | 0.383906 | 0.296762 | 0.458655 | 0.306473 | 0.337856 | 0.311131 | 0.338069 | 0.378135 |
| LEU22 | 0.505377 | 0.371704 | 0.27037 | 0.449691 | 0.447388 | 0.426572 | 0.438615 | 0.461042 | 0.287163 | 0.304284 | 0.287084 | 0.506686 | 0.276204 | 0.380371 | 0.402739 | 0.371536 | 0.356118 |
| PHE23 | 0.427942 | 0.388695 | 0.316205 | 0.515901 | 0.377779 | 0.375709 | 0.500803 | 0.413976 | 0.376544 | 0.309105 | 0.308272 | 0.551741 | 0.278927 | 0.358465 | 0.579404 | 0.508009 | 0.42385 |
| VAL24 | 0.359066 | 0.434045 | 0.388151 | 0.401451 | 0.345259 | 0.356334 | 0.467308 | 0.323812 | 0.372781 | 0.502887 | 0.364461 | 0.494483 | 0.289139 | 0.407819 | 0.362614 | 0.46884 | 0.496186 |
| PHE25 | 0.533179 | 0.414634 | 0.430894 | 0.465594 | 0.594363 | 0.534456 | 0.525094 | 0.431175 | 0.48331 | 0.508996 | 0.605112 | 0.520954 | 0.386631 | 0.412316 | 0.562384 | 0.347668 | 0.487587 |
| PHE26 | 0.482362 | 0.560274 | 0.678226 | 0.773259 | 0.606175 | 0.468973 | 0.552193 | 0.441676 | 0.660747 | 0.682001 | 0.479834 | 0.650969 | 0.388871 | 0.306584 | 0.564009 | 0.374934 | 0.614681 |
| GLY27 | 0.359772 | 0.340068 | 0.470943 | 0.326404 | 0.39536 | 0.283241 | 0.398273 | 0.417096 | 0.553981 | 0.613881 | 0.244037 | 0.587249 | 0.31504 | 0.385343 | 0.411915 | 0.361774 | 0.461497 |
| LEU28 | 0.422772 | 0.280815 | 0.444995 | 0.401695 | 0.342213 | 0.416665 | 0.49584 | 0.388861 | 0.527755 | 0.617537 | 0.328095 | 0.672859 | 0.404795 | 0.341657 | 0.553086 | 0.341652 | 0.343242 |
| GLY29 | 0.4025 | 0.263768 | 0.328272 | 0.44806 | 0.401248 | 0.44411 | 0.373256 | 0.409627 | 0.55879 | 0.432618 | 0.286776 | 0.486887 | 0.428057 | 0.327654 | 0.342524 | 0.358794 | 0.389392 |
| SER30 | 0.366187 | 0.264703 | 0.393026 | 0.439509 | 0.451138 | 0.408586 | 0.47207 | 0.509779 | 0.428158 | 0.582509 | 0.391158 | 0.418105 | 0.445185 | 0.489988 | 0.254396 | 0.473762 | 0.399373 |
| ALA31 | 0.476516 | 0.262545 | 0.314128 | 0.436934 | 0.455678 | 0.482672 | 0.521546 | 0.449883 | 0.403964 | 0.64273 | 0.365963 | 0.688064 | 0.474431 | 0.450491 | 0.37807 | 0.418892 | 0.553955 |
| LEU32 | 0.532018 | 0.350262 | 0.427495 | 0.4589 | 0.426103 | 0.545603 | 0.642558 | 0.409331 | 0.447966 | 0.562592 | 0.280447 | 0.632218 | 0.38975 | 0.476727 | 0.529333 | 0.460015 | 0.545458 |
| ASN33 | 0.481526 | 0.397569 | 0.502421 | 0.665215 | 0.495413 | 0.685659 | 1.36015 | 0.655163 | 0.787203 | 0.517685 | 0.370239 | 0.8855 | 0.495135 | 0.488118 | 0.471393 | 0.547607 | 0.608147 |
| TRP34 | 0.955914 | 0.492213 | 0.553012 | 0.446515 | 0.525096 | 0.563455 | 0.759134 | 0.578929 | 0.509116 | 0.801297 | 0.377295 | 0.52908 | 0.522014 | 0.530101 | 0.580152 | 0.499799 | 0.452388 |
| PRO35 | 0.78246 | 0.508134 | 0.546583 | 0.30205 | 0.388539 | 0.682553 | 0.866834 | 0.691776 | 0.62035 | 0.777325 | 0.600313 | 0.553757 | 0.615162 | 0.430769 | 0.645663 | 0.567332 | 0.559918 |
| GLN36 | 0.925996 | 0.707986 | 0.670275 | 0.564867 | 0.786775 | 0.889171 | 1.03625 | 0.836904 | 0.712887 | 1.00637 | 0.913053 | 0.556076 | 1.00108 | 0.765058 | 0.818445 | 0.786848 | 1.00601 |
| ALA37 | 0.623277 | 0.611219 | 0.545728 | 0.535964 | 0.939824 | 0.758123 | 0.665147 | 0.667839 | 0.959701 | 0.798882 | 0.400547 | 0.456046 | 0.755657 | 0.640269 | 0.841715 | 0.596752 | 0.862498 |
| LEU38 | 0.577279 | 0.665214 | 0.560332 | 0.423857 | 0.858086 | 0.660537 | 0.844829 | 0.60278 | 0.781449 | 0.827757 | 0.45908 | 0.609968 | 0.912385 | 0.691957 | 0.589684 | 0.726618 | 1.04809 |
| PRO39 | 0.424446 | 0.350227 | 0.58259 | 0.463482 | 0.493384 | 0.574814 | 0.622743 | 0.612745 | 0.546145 | 0.731608 | 0.365603 | 0.535123 | 0.730202 | 0.651795 | 0.583915 | 0.574196 | 0.799294 |
| SER40 | 0.390197 | 0.417848 | 0.445844 | 0.490324 | 0.553722 | 0.556653 | 0.598751 | 0.556677 | 0.656137 | 0.581656 | 0.601192 | 0.522634 | 0.64429 | 0.676818 | 0.636818 | 0.384034 | 0.577123 |
| VAL41 | 0.591095 | 0.544706 | 0.694157 | 0.541215 | 0.512491 | 0.486953 | 0.643093 | 0.503728 | 0.456725 | 0.410257 | 0.512157 | 0.445928 | 0.62783 | 0.501646 | 0.523787 | 0.344877 | 0.528172 |
| LEU42 | 0.612567 | 0.608019 | 0.530809 | 0.559678 | 0.594913 | 0.77369 | 0.648564 | 0.44004 | 0.524112 | 0.412022 | 0.54379 | 0.604347 | 0.570964 | 0.583628 | 0.513319 | 0.495774 | 0.607247 |
| GLN43 | 0.317367 | 0.454989 | 0.553171 | 0.655414 | 0.589967 | 0.752057 | 0.800893 | 0.53374 | 0.553566 | 0.499842 | 0.67951 | 0.391459 | 0.501742 | 0.521971 | 0.426211 | 0.488348 | 0.734436 |
| ILE44 | 0.379184 | 0.348539 | 0.517269 | 0.437298 | 0.488465 | 0.503888 | 0.430323 | 0.536095 | 0.360864 | 0.478781 | 0.360517 | 0.320817 | 0.464449 | 0.535698 | 0.463915 | 0.389649 | 0.497263 |
| ALA45 | 0.521715 | 0.445287 | 0.50339 | 0.37589 | 0.414774 | 0.456409 | 0.502125 | 0.506069 | 0.432578 | 0.347057 | 0.370184 | 0.394956 | 0.367796 | 0.42155 | 0.4516 | 0.334164 | 0.41764 |
| MET46 | 0.607906 | 0.721053 | 0.621883 | 0.58303 | 0.631835 | 0.450333 | 0.627795 | 0.598578 | 0.525342 | 0.566167 | 0.504662 | 0.539254 | 0.522196 | 0.630483 | 0.626458 | 0.695948 | 0.569592 |
| ALA47 | 0.519376 | 0.472247 | 0.414479 | 0.32439 | 0.436298 | 0.339725 | 0.580843 | 0.512282 | 0.369824 | 0.458814 | 0.33639 | 0.394246 | 0.398027 | 0.354556 | 0.461947 | 0.432597 | 0.403225 |
| PHE48 | 0.626736 | 0.53349 | 0.520152 | 0.37391 | 0.386418 | 0.285071 | 0.494625 | 0.471912 | 0.47303 | 0.318138 | 0.382874 | 0.46624 | 0.332529 | 0.441165 | 0.352682 | 0.434548 | 0.467906 |
| GLY49 | 0.509927 | 0.429689 | 0.2825 | 0.35526 | 0.414869 | 0.376028 | 0.420579 | 0.426422 | 0.424203 | 0.318643 | 0.391735 | 0.532482 | 0.250473 | 0.402842 | 0.474418 | 0.4894 | 0.514455 |
| LEU50 | 0.546966 | 0.473555 | 0.329008 | 0.413285 | 0.438837 | 0.504003 | 0.488545 | 0.463596 | 0.66317 | 0.600522 | 0.454437 | 0.607607 | 0.424448 | 0.615353 | 0.636664 | 0.834776 | 0.576116 |
| GLY51 | 0.617299 | 0.583311 | 0.317964 | 0.370694 | 0.328491 | 0.552597 | 0.510449 | 0.358043 | 0.502495 | 0.457146 | 0.450884 | 0.440053 | 0.468514 | 0.428935 | 0.431171 | 0.588992 | 0.675951 |
| ILE52 | 0.635091 | 0.440711 | 0.341188 | 0.364614 | 0.423034 | 0.407442 | 0.452726 | 0.393123 | 0.374752 | 0.517519 | 0.375012 | 0.55501 | 0.401109 | 0.489575 | 0.424752 | 0.495389 | 0.493978 |
| GLY53 | 0.535375 | 0.362933 | 0.299233 | 0.444886 | 0.340743 | 0.479376 | 0.578167 | 0.353706 | 0.454744 | 0.356447 | 0.315157 | 0.584823 | 0.488891 | 0.484281 | 0.503752 | 0.444194 | 0.446734 |
| THR54 | 0.528435 | 0.46394 | 0.381556 | 0.438594 | 0.2623 | 0.531013 | 0.479935 | 0.296177 | 0.434466 | 0.424898 | 0.378751 | 0.480444 | 0.43094 | 0.448876 | 0.468289 | 0.433905 | 0.52008 |
| LEU55 | 0.646059 | 0.339435 | 0.444404 | 0.503362 | 0.299693 | 0.495164 | 0.542588 | 0.438477 | 0.419837 | 0.408003 | 0.253049 | 0.434218 | 0.346127 | 0.395403 | 0.283045 | 0.525076 | 0.39633 |
| VAL56 | 0.598026 | 0.435105 | 0.562667 | 0.569803 | 0.345983 | 0.423077 | 0.541735 | 0.615185 | 0.433247 | 0.383599 | 0.399208 | 0.355504 | 0.463051 | 0.364676 | 0.328206 | 0.42929 | 0.414322 |
| GLN57 | 0.557289 | 0.470938 | 0.641119 | 0.6743 | 0.369909 | 0.518313 | 0.63984 | 0.578747 | 0.557103 | 0.423429 | 0.607495 | 0.603422 | 0.609096 | 0.468451 | 0.502657 | 0.630548 | 0.431449 |
| ALA58 | 0.495299 | 0.413807 | 0.481933 | 0.609552 | 0.374342 | 0.668826 | 0.630815 | 0.409282 | 0.439689 | 0.359961 | 0.304161 | 0.557646 | 0.502525 | 0.589732 | 0.40566 | 0.665247 | 0.431709 |
| LEU59 | 0.464715 | 0.468494 | 0.468776 | 0.378804 | 0.386859 | 0.585931 | 0.705731 | 0.497065 | 0.444001 | 0.463063 | 0.314202 | 0.533355 | 0.56625 | 0.46667 | 0.406675 | 0.778945 | 0.557982 |
| GLY60 | 0.424343 | 0.458096 | 0.500836 | 0.462714 | 0.32659 | 0.47319 | 0.857573 | 0.613452 | 0.435388 | 0.376989 | 0.411009 | 0.70575 | 0.424741 | 0.499026 | 0.403681 | 0.676828 | 0.543427 |
| HIS61 | 0.738429 | 0.45512 | 0.666347 | 0.481386 | 0.674375 | 0.762541 | 0.803146 | 0.676706 | 0.324418 | 0.382206 | 0.583567 | 0.611115 | 0.67023 | 0.840061 | 0.656196 | 0.526482 | 0.578471 |
| ILE62 | 0.37864 | 0.382922 | 0.652806 | 0.329361 | 0.453265 | 0.565974 | 0.545046 | 0.565816 | 0.429962 | 0.344486 | 0.546913 | 0.502809 | 0.477849 | 0.508011 | 0.437338 | 0.566897 | 0.457781 |
| SER63 | 0.3529 | 0.417029 | 0.471255 | 0.364486 | 0.492291 | 0.500909 | 0.471722 | 0.450516 | 0.356016 | 0.488851 | 0.531602 | 0.605615 | 0.465399 | 0.449572 | 0.428496 | 0.383057 | 0.582919 |
| GLY64 | 0.481022 | 0.37449 | 0.385159 | 0.665927 | 0.330282 | 0.247075 | 0.506111 | 0.528854 | 0.355395 | 0.298091 | 0.442579 | 0.7894 | 0.620392 | 0.423212 | 0.478312 | 0.500517 | 0.441894 |
| ALA65 | 0.516465 | 0.336447 | 0.413609 | 0.586403 | 0.334511 | 0.409191 | 0.506554 | 0.549839 | 0.505325 | 0.329124 | 0.539516 | 0.627758 | 0.537504 | 0.54151 | 0.417938 | 0.500557 | 0.458594 |
| HIS66 | 0.636385 | 0.383087 | 0.402883 | 0.542192 | 0.361667 | 0.384909 | 0.365839 | 0.428772 | 0.369832 | 0.401708 | 0.360411 | 0.50715 | 0.451735 | 0.374601 | 0.395764 | 0.274855 | 0.353676 |
| ILE67 | 0.489319 | 0.31702 | 0.37306 | 0.39639 | 0.354272 | 0.292019 | 0.389759 | 0.409365 | 0.315688 | 0.247465 | 0.312813 | 0.475315 | 0.467587 | 0.261272 | 0.366277 | 0.378006 | 0.381834 |
| ASN68 | 0.473044 | 0.246648 | 0.326664 | 0.334579 | 0.351669 | 0.384015 | 0.298683 | 0.307871 | 0.317147 | 0.273242 | 0.328188 | 0.42089 | 0.475838 | 0.28713 | 0.266052 | 0.257556 | 0.280826 |
| PRO69 | 0.529056 | 0.297114 | 0.258713 | 0.37616 | 0.372889 | 0.418672 | 0.34129 | 0.314407 | 0.385287 | 0.187239 | 0.576642 | 0.445461 | 0.338362 | 0.334609 | 0.511721 | 0.272098 | 0.414647 |
| ALA70 | 0.534471 | 0.275616 | 0.232645 | 0.644588 | 0.412166 | 0.294324 | 0.333569 | 0.344893 | 0.350272 | 0.270957 | 0.541496 | 0.50515 | 0.319215 | 0.305007 | 0.384692 | 0.362754 | 0.385509 |
| VAL71 | 0.656041 | 0.278367 | 0.272783 | 0.690058 | 0.421724 | 0.34426 | 0.313607 | 0.314952 | 0.319454 | 0.295439 | 0.597926 | 0.388086 | 0.393857 | 0.320869 | 0.245916 | 0.396524 | 0.367782 |
| THR72 | 0.391974 | 0.293784 | 0.344348 | 0.328356 | 0.360583 | 0.365733 | 0.2783 | 0.324669 | 0.340195 | 0.365452 | 0.337245 | 0.402475 | 0.399699 | 0.292013 | 0.22151 | 0.273021 | 0.350439 |
| VAL73 | 0.433016 | 0.299643 | 0.264879 | 0.461292 | 0.49381 | 0.279271 | 0.369171 | 0.371603 | 0.435882 | 0.48377 | 0.377868 | 0.497735 | 0.331092 | 0.359213 | 0.269653 | 0.39754 | 0.388971 |
| ALA74 | 0.519035 | 0.334911 | 0.225666 | 0.455889 | 0.423344 | 0.248773 | 0.42169 | 0.364967 | 0.396223 | 0.377161 | 0.41768 | 0.50033 | 0.306909 | 0.410965 | 0.329187 | 0.296343 | 0.429228 |
| CYS75 | 0.484079 | 0.342558 | 0.346245 | 0.434874 | 0.42441 | 0.318281 | 0.418239 | 0.508101 | 0.291707 | 0.402729 | 0.341539 | 0.443217 | 0.446039 | 0.473884 | 0.434944 | 0.386911 | 0.494644 |
| LEU76 | 0.383219 | 0.279293 | 0.466705 | 0.682674 | 0.548965 | 0.372659 | 0.401504 | 0.414971 | 0.338435 | 0.401874 | 0.297071 | 0.470138 | 0.364447 | 0.524633 | 0.424273 | 0.509984 | 0.416553 |
| VAL77 | 0.348132 | 0.296192 | 0.472104 | 0.668727 | 0.452915 | 0.480262 | 0.566772 | 0.598453 | 0.403963 | 0.409078 | 0.37085 | 0.437167 | 0.506371 | 0.467121 | 0.560709 | 0.35343 | 0.513022 |
| GLY78 | 0.575685 | 0.287703 | 0.469541 | 0.676853 | 0.371861 | 0.437264 | 0.476352 | 0.651436 | 0.400542 | 0.462528 | 0.344491 | 0.38184 | 0.544556 | 0.471685 | 0.531211 | 0.409646 | 0.584003 |
| CYS79 | 0.705339 | 0.44871 | 0.633507 | 0.709822 | 0.415989 | 0.546572 | 0.688774 | 0.597349 | 0.585127 | 0.653193 | 0.485854 | 0.446614 | 0.514888 | 0.817729 | 0.714855 | 0.706341 | 0.685353 |
| HIS80 | 0.707384 | 0.598454 | 0.596289 | 0.545935 | 0.484486 | 0.63309 | 0.586322 | 0.611501 | 0.818519 | 0.613615 | 0.368745 | 0.508284 | 0.537815 | 0.702389 | 0.566595 | 0.399839 | 0.824952 |
| VAL81 | 0.63697 | 0.528538 | 0.542444 | 0.445644 | 0.388476 | 0.294485 | 0.338202 | 0.407297 | 0.37191 | 0.484108 | 0.494939 | 0.31011 | 0.503803 | 0.634029 | 0.493327 | 0.540489 | 0.493538 |
| SER82 | 0.626696 | 0.558319 | 0.534823 | 0.651888 | 0.385121 | 0.304248 | 0.352298 | 0.385083 | 0.372167 | 0.388476 | 0.379758 | 0.404398 | 0.461048 | 0.486573 | 0.566446 | 0.459447 | 0.465392 |
| VAL83 | 0.65044 | 0.570517 | 0.585649 | 0.55672 | 0.404323 | 0.403312 | 0.429288 | 0.41538 | 0.444124 | 0.421422 | 0.354498 | 0.488481 | 0.29016 | 0.440281 | 0.595484 | 0.464609 | 0.434593 |
| LEU84 | 0.670289 | 0.654801 | 0.547273 | 0.389366 | 0.724028 | 0.389071 | 0.433188 | 0.475776 | 0.466215 | 0.509223 | 0.425657 | 0.584647 | 0.493907 | 0.729513 | 0.5682 | 0.356338 | 0.561284 |
| ARG85 | 0.545254 | 0.378996 | 0.486138 | 0.706888 | 0.420536 | 0.301533 | 0.3617 | 0.508846 | 0.350969 | 0.328021 | 0.379678 | 0.429277 | 0.336242 | 0.459458 | 0.381265 | 0.400913 | 0.588956 |
| ALA86 | 0.555645 | 0.39105 | 0.431751 | 0.307811 | 0.320857 | 0.258786 | 0.223057 | 0.324729 | 0.311168 | 0.295083 | 0.323142 | 0.406019 | 0.349015 | 0.378578 | 0.414247 | 0.438454 | 0.45812 |
| ALA87 | 0.526998 | 0.519744 | 0.461769 | 0.394908 | 0.364182 | 0.44374 | 0.30229 | 0.563888 | 0.462096 | 0.388391 | 0.401928 | 0.420961 | 0.413981 | 0.374158 | 0.406761 | 0.439519 | 0.457757 |
| PHE88 | 0.413476 | 0.594369 | 0.457388 | 0.477958 | 0.577673 | 0.402958 | 0.394649 | 0.517547 | 0.42084 | 0.48581 | 0.397826 | 0.587459 | 0.658199 | 0.43541 | 0.500018 | 0.405354 | 0.655478 |
| TYR89 | 0.470218 | 0.316394 | 0.519826 | 0.430777 | 0.327545 | 0.402634 | 0.432429 | 0.556824 | 0.361213 | 0.280161 | 0.311224 | 0.569651 | 0.370149 | 0.353758 | 0.417387 | 0.259832 | 0.545095 |
| VAL90 | 0.411415 | 0.330445 | 0.401788 | 0.286035 | 0.358607 | 0.421926 | 0.444649 | 0.232295 | 0.402687 | 0.402548 | 0.287809 | 0.566853 | 0.222176 | 0.340248 | 0.394217 | 0.276818 | 0.386217 |
| ALA91 | 0.337737 | 0.366914 | 0.311113 | 0.305094 | 0.348951 | 0.395254 | 0.304977 | 0.27422 | 0.388928 | 0.271851 | 0.403912 | 0.596484 | 0.337838 | 0.412212 | 0.355084 | 0.267015 | 0.369152 |
| ALA92 | 0.39244 | 0.35513 | 0.290512 | 0.291872 | 0.276821 | 0.304173 | 0.280659 | 0.310602 | 0.301903 | 0.299689 | 0.364245 | 0.55299 | 0.326472 | 0.342221 | 0.358954 | 0.25532 | 0.417888 |
| GLN93 | 0.313654 | 0.259694 | 0.331586 | 0.326711 | 0.301963 | 0.397557 | 0.331042 | 0.307808 | 0.33905 | 0.333286 | 0.351942 | 0.478752 | 0.275887 | 0.282899 | 0.316542 | 0.272695 | 0.309707 |
| LEU94 | 0.354771 | 0.294611 | 0.41824 | 0.285895 | 0.394713 | 0.571134 | 0.311555 | 0.389194 | 0.541068 | 0.326597 | 0.523488 | 0.475375 | 0.364352 | 0.315657 | 0.371348 | 0.344152 | 0.428907 |
| LEU95 | 0.418872 | 0.222462 | 0.577896 | 0.402031 | 0.439648 | 0.489219 | 0.415306 | 0.380401 | 0.418716 | 0.483198 | 0.465034 | 0.491769 | 0.376663 | 0.287502 | 0.37804 | 0.442279 | 0.532165 |
| GLY96 | 0.237606 | 0.18124 | 0.361283 | 0.24408 | 0.283037 | 0.388634 | 0.374116 | 0.355595 | 0.465392 | 0.398671 | 0.312543 | 0.330531 | 0.364444 | 0.183338 | 0.333546 | 0.216071 | 0.343793 |
| ALA97 | 0.318867 | 0.202312 | 0.330428 | 0.285904 | 0.27942 | 0.440318 | 0.399112 | 0.356668 | 0.39371 | 0.390069 | 0.26606 | 0.414759 | 0.375498 | 0.249119 | 0.363968 | 0.259571 | 0.29718 |
| VAL98 | 0.587643 | 0.261119 | 0.391563 | 0.299224 | 0.446555 | 0.460076 | 0.360233 | 0.277147 | 0.3243 | 0.42389 | 0.374212 | 0.272905 | 0.4735 | 0.364253 | 0.341801 | 0.263309 | 0.48451 |
| ALA99 | 0.452556 | 0.234235 | 0.428335 | 0.312328 | 0.46806 | 0.448398 | 0.288072 | 0.302262 | 0.49876 | 0.452775 | 0.44778 | 0.315092 | 0.530976 | 0.282569 | 0.349553 | 0.299868 | 0.548853 |
| GLY100 | 0.336698 | 0.276559 | 0.52758 | 0.313355 | 0.46111 | 0.395426 | 0.407701 | 0.284611 | 0.328578 | 0.422232 | 0.404441 | 0.348549 | 0.470845 | 0.297656 | 0.287377 | 0.329801 | 0.396422 |
| ALA101 | 0.512332 | 0.265664 | 0.406491 | 0.441883 | 0.402916 | 0.352869 | 0.402514 | 0.30692 | 0.350501 | 0.357207 | 0.352789 | 0.360591 | 0.352569 | 0.381026 | 0.344838 | 0.384681 | 0.386096 |
| ALA102 | 0.513812 | 0.24999 | 0.450805 | 0.364409 | 0.588833 | 0.510662 | 0.457197 | 0.29894 | 0.46376 | 0.412532 | 0.454923 | 0.371183 | 0.33168 | 0.387481 | 0.34757 | 0.424489 | 0.487353 |
| LEU103 | 0.499564 | 0.42509 | 0.651394 | 0.57734 | 0.703895 | 0.710305 | 0.45972 | 0.323903 | 0.480486 | 0.623483 | 0.496875 | 0.616945 | 0.554871 | 0.48836 | 0.504147 | 0.429248 | 0.509577 |
| LEU104 | 0.4633 | 0.324732 | 0.450141 | 0.426109 | 0.449471 | 0.504971 | 0.480674 | 0.376192 | 0.400353 | 0.420458 | 0.294946 | 0.436849 | 0.448475 | 0.390671 | 0.392919 | 0.404535 | 0.442322 |
| HIS105 | 0.492051 | 0.418299 | 0.537303 | 0.415595 | 0.620284 | 0.659079 | 0.500669 | 0.492269 | 0.461216 | 0.510294 | 0.440898 | 0.403119 | 0.487151 | 0.545003 | 0.530245 | 0.614175 | 0.553714 |
| GLU106 | 0.794576 | 0.641645 | 0.979245 | 0.477866 | 0.798328 | 0.939885 | 0.594605 | 0.646081 | 0.63323 | 0.596083 | 0.729606 | 0.575778 | 0.671334 | 0.597102 | 0.659357 | 0.565051 | 0.608648 |
| ILE107 | 0.546243 | 0.426195 | 0.733695 | 0.387083 | 0.636494 | 0.884113 | 0.541337 | 0.451925 | 0.536444 | 0.567269 | 0.513212 | 0.626895 | 0.491335 | 0.55519 | 0.512303 | 0.477826 | 0.601232 |
| THR108 | 0.414079 | 0.481263 | 0.557644 | 0.299362 | 0.392634 | 0.655085 | 0.50277 | 0.416825 | 0.501869 | 0.539804 | 0.469777 | 0.480928 | 0.497983 | 0.447746 | 0.441485 | 0.451047 | 0.659198 |
| PRO109 | 0.596874 | 0.415592 | 0.596938 | 0.431834 | 0.40812 | 0.712074 | 0.520704 | 0.455941 | 0.699824 | 0.562933 | 0.450741 | 0.511099 | 0.69095 | 0.533608 | 0.537018 | 0.499368 | 0.641313 |
| ALA110 | 0.897012 | 0.529355 | 0.550821 | 0.409434 | 0.43271 | 0.721875 | 0.510849 | 0.41911 | 0.825869 | 0.51817 | 0.520799 | 0.659456 | 0.837303 | 0.62957 | 0.552385 | 0.545192 | 0.691264 |
| ASP111 | 1.21778 | 0.765693 | 0.757535 | 0.430097 | 0.694927 | 0.956075 | 0.919344 | 0.590613 | 0.707527 | 0.659621 | 0.875914 | 0.937955 | 0.783732 | 0.598528 | 0.507507 | 0.662561 | 0.739778 |
| ILE112 | 0.701241 | 0.614098 | 0.626217 | 0.745891 | 0.570658 | 0.740318 | 0.615733 | 0.614077 | 0.694976 | 0.577438 | 0.54912 | 0.625278 | 0.546815 | 0.515594 | 0.52171 | 0.553291 | 0.571889 |
| ARG113 | 0.74395 | 0.521422 | 0.509799 | 0.444945 | 0.396449 | 0.674906 | 0.491903 | 0.457121 | 0.65012 | 0.454171 | 0.464244 | 0.534689 | 0.531758 | 0.49719 | 0.496975 | 0.475327 | 0.640147 |
| GLY114 | 0.688389 | 0.496995 | 0.370136 | 0.629201 | 0.495811 | 0.693464 | 0.501707 | 0.524611 | 0.707418 | 0.478471 | 0.503693 | 0.557892 | 0.570055 | 0.379131 | 0.454512 | 0.607828 | 0.517105 |
| ASP115 | 0.593034 | 0.612235 | 0.334654 | 0.457551 | 0.413617 | 0.772248 | 0.456383 | 0.458929 | 0.677271 | 0.442021 | 0.453145 | 0.667896 | 0.354888 | 0.430624 | 0.453224 | 0.53419 | 0.628903 |
| LEU116 | 0.448363 | 0.507067 | 0.299197 | 0.359812 | 0.609452 | 0.480063 | 0.460504 | 0.546185 | 0.526667 | 0.47766 | 0.523594 | 0.537618 | 0.429453 | 0.44865 | 0.439756 | 0.599873 | 0.407724 |
| ALA117 | 0.410443 | 0.406019 | 0.356829 | 0.289221 | 0.447648 | 0.264346 | 0.356218 | 0.504185 | 0.497425 | 0.356842 | 0.391945 | 0.483198 | 0.40424 | 0.40426 | 0.412761 | 0.417013 | 0.399249 |
| VAL118 | 0.571302 | 0.417756 | 0.471594 | 0.443463 | 0.558048 | 0.349231 | 0.431707 | 0.573605 | 0.5242 | 0.386787 | 0.407809 | 0.49461 | 0.333364 | 0.388526 | 0.371896 | 0.547673 | 0.514354 |
| ASN119 | 0.369624 | 0.553074 | 0.413435 | 0.295946 | 0.401347 | 0.502524 | 0.612795 | 0.431366 | 0.512689 | 0.451169 | 0.500013 | 0.450068 | 0.483764 | 0.544164 | 0.404362 | 0.476581 | 0.450117 |
| ALA120 | 0.486747 | 0.494459 | 0.387048 | 0.411091 | 0.486427 | 0.544664 | 0.427283 | 0.441647 | 0.531307 | 0.579708 | 0.463208 | 0.600144 | 0.731939 | 0.571987 | 0.504353 | 0.514809 | 0.720334 |
| LEU121 | 0.564467 | 0.564055 | 0.681407 | 0.487732 | 0.58554 | 0.522205 | 0.521439 | 0.449378 | 0.559065 | 0.37525 | 0.746793 | 0.727173 | 0.59158 | 0.508845 | 0.603737 | 0.572121 | 0.411273 |
| SER122 | 0.600673 | 0.428138 | 0.469491 | 0.496981 | 0.489601 | 0.422796 | 0.473445 | 0.676083 | 0.55597 | 0.405846 | 0.625611 | 0.775908 | 0.449906 | 0.412144 | 0.624488 | 0.827637 | 0.501379 |
| ASN123 | 0.751395 | 0.661084 | 0.584866 | 0.720347 | 0.816553 | 0.684502 | 0.586595 | 0.546302 | 0.870896 | 0.482357 | 0.830836 | 0.712759 | 0.507777 | 0.505736 | 0.900429 | 0.972306 | 0.735948 |
| SER124 | 0.788886 | 0.739683 | 0.709265 | 0.603281 | 0.771983 | 0.782006 | 0.71495 | 0.81963 | 0.975947 | 0.466652 | 0.517266 | 0.69276 | 0.540918 | 0.509893 | 0.798889 | 1.09932 | 0.520772 |
| THR125 | 0.683899 | 0.550321 | 0.606668 | 0.450599 | 0.447753 | 0.547076 | 0.43657 | 0.583798 | 0.647773 | 0.455122 | 0.365883 | 0.527049 | 0.585607 | 0.417546 | 0.695072 | 0.712826 | 0.404712 |
| THR126 | 0.583472 | 0.63204 | 0.511603 | 0.495819 | 0.381331 | 0.445638 | 0.570416 | 0.423277 | 0.688054 | 0.52147 | 0.431077 | 0.62479 | 0.847985 | 0.633395 | 0.538105 | 0.632143 | 0.549299 |
| ALA127 | 0.596026 | 0.449954 | 0.412212 | 0.419288 | 0.409702 | 0.364274 | 0.48859 | 0.419841 | 0.671089 | 0.350454 | 0.49285 | 0.504525 | 0.939521 | 0.546937 | 0.723094 | 0.47016 | 0.536986 |
| GLY128 | 0.623874 | 0.543032 | 0.44717 | 0.528723 | 0.491838 | 0.456543 | 0.605888 | 0.36395 | 0.522104 | 0.348352 | 0.487989 | 0.500531 | 1.01527 | 0.510473 | 0.618485 | 0.577789 | 0.572395 |
| GLN129 | 0.842746 | 0.803946 | 0.560927 | 0.569481 | 0.580452 | 0.622912 | 0.644691 | 0.403129 | 0.515351 | 0.412869 | 0.498674 | 0.661307 | 0.678512 | 0.563514 | 0.371174 | 0.610147 | 0.715513 |
| ALA130 | 0.398424 | 0.454782 | 0.373497 | 0.405054 | 0.286758 | 0.354472 | 0.311159 | 0.274893 | 0.418989 | 0.222162 | 0.377205 | 0.349863 | 0.381778 | 0.383203 | 0.341087 | 0.264157 | 0.401134 |
| VAL131 | 0.364396 | 0.429706 | 0.518029 | 0.397177 | 0.39678 | 0.439734 | 0.425248 | 0.28242 | 0.446696 | 0.258528 | 0.415861 | 0.350254 | 0.465642 | 0.474371 | 0.387416 | 0.250139 | 0.475928 |
| THR132 | 0.367489 | 0.513728 | 0.297909 | 0.347656 | 0.441764 | 0.627215 | 0.479956 | 0.318454 | 0.797108 | 0.273635 | 0.518059 | 0.535518 | 0.707067 | 0.455168 | 0.460969 | 0.27381 | 0.499414 |
| VAL133 | 0.368789 | 0.474123 | 0.286094 | 0.379598 | 0.337446 | 0.421927 | 0.38988 | 0.293612 | 0.589108 | 0.408775 | 0.47389 | 0.39572 | 0.504995 | 0.361477 | 0.430592 | 0.303104 | 0.368187 |
| GLU134 | 0.336793 | 0.46106 | 0.312322 | 0.27491 | 0.303658 | 0.386672 | 0.345526 | 0.283352 | 0.34466 | 0.262901 | 0.317603 | 0.254375 | 0.411035 | 0.386185 | 0.355885 | 0.285842 | 0.377368 |
| LEU135 | 0.31792 | 0.513735 | 0.324683 | 0.381691 | 0.406814 | 0.451972 | 0.584329 | 0.276412 | 0.509938 | 0.266495 | 0.381934 | 0.310296 | 0.591411 | 0.353726 | 0.443185 | 0.438339 | 0.555282 |
| PHE136 | 0.338183 | 0.471897 | 1.06493 | 0.516332 | 0.652312 | 0.546061 | 0.415755 | 0.438914 | 0.504924 | 0.366825 | 0.539169 | 0.441604 | 0.792227 | 0.493248 | 0.568069 | 0.510124 | 0.509898 |
| LEU137 | 0.386228 | 0.489726 | 0.436692 | 0.417003 | 0.41125 | 0.403733 | 0.342138 | 0.280445 | 0.409746 | 0.362633 | 0.266686 | 0.466309 | 0.579158 | 0.325743 | 0.304547 | 0.439109 | 0.547264 |
| THR138 | 0.23016 | 0.316664 | 0.4476 | 0.371474 | 0.239516 | 0.358049 | 0.376195 | 0.303682 | 0.306229 | 0.303863 | 0.269204 | 0.410006 | 0.444176 | 0.383977 | 0.239316 | 0.332093 | 0.443445 |
| LEU139 | 0.303663 | 0.340838 | 0.450456 | 0.533138 | 0.506067 | 0.458068 | 0.568643 | 0.42163 | 0.403659 | 0.366795 | 0.498189 | 0.403062 | 0.572202 | 0.384983 | 0.389973 | 0.403713 | 0.614283 |
| GLN140 | 0.267092 | 0.455859 | 0.428751 | 0.421375 | 0.394502 | 0.511848 | 0.517764 | 0.457509 | 0.543861 | 0.462512 | 0.480683 | 0.45333 | 0.702366 | 0.442621 | 0.523192 | 0.481657 | 0.560532 |
| LEU141 | 0.291786 | 0.382523 | 0.39631 | 0.420584 | 0.314405 | 0.331722 | 0.392309 | 0.376285 | 0.501327 | 0.323561 | 0.4262 | 0.547917 | 0.705459 | 0.425757 | 0.388666 | 0.404388 | 0.433375 |
| VAL142 | 0.30178 | 0.347823 | 0.340174 | 0.37364 | 0.369911 | 0.51167 | 0.493924 | 0.405925 | 0.390231 | 0.427932 | 0.567423 | 0.476262 | 0.567277 | 0.375285 | 0.459225 | 0.359511 | 0.44888 |
| LEU143 | 0.405563 | 0.472133 | 0.38206 | 0.459408 | 0.401497 | 0.57705 | 0.561881 | 0.396124 | 0.369108 | 0.474977 | 0.6897 | 0.644279 | 0.4591 | 0.457247 | 0.411428 | 0.392254 | 0.563789 |
| CYS144 | 0.318507 | 0.447661 | 0.431976 | 0.380776 | 0.293072 | 0.432238 | 0.352238 | 0.311973 | 0.444626 | 0.329382 | 0.373105 | 0.421599 | 0.556062 | 0.380261 | 0.316693 | 0.286868 | 0.342743 |
| ILE145 | 0.412228 | 0.303072 | 0.409109 | 0.395885 | 0.330463 | 0.394944 | 0.410911 | 0.345844 | 0.383734 | 0.321139 | 0.250933 | 0.405056 | 0.463167 | 0.396544 | 0.338103 | 0.371839 | 0.425368 |
| PHE146 | 0.557032 | 0.519274 | 0.443962 | 0.317179 | 0.337182 | 0.380617 | 0.609767 | 0.470459 | 0.597291 | 0.357769 | 0.344519 | 0.663699 | 0.545741 | 0.394335 | 0.565837 | 0.451154 | 0.537562 |
| ALA147 | 0.385054 | 0.427781 | 0.364751 | 0.286856 | 0.317694 | 0.505015 | 0.519547 | 0.407364 | 0.390309 | 0.411035 | 0.309824 | 0.601739 | 0.452788 | 0.340648 | 0.515224 | 0.47216 | 0.398256 |
| SER148 | 0.514449 | 0.356929 | 0.434499 | 0.420389 | 0.474755 | 0.682314 | 0.446614 | 0.368886 | 0.373458 | 0.477907 | 0.334199 | 0.41205 | 0.525587 | 0.440345 | 0.5803 | 0.37827 | 0.420963 |
| THR149 | 0.61867 | 0.409432 | 0.513286 | 0.378319 | 0.454245 | 0.428123 | 0.440952 | 0.43482 | 0.366346 | 0.374237 | 0.29281 | 0.649369 | 0.537967 | 0.515774 | 0.432288 | 0.401477 | 0.63261 |
| ASP150 | 0.564919 | 0.643581 | 0.54043 | 0.326988 | 0.350358 | 0.717663 | 0.399343 | 0.506218 | 0.431468 | 0.353466 | 0.426083 | 0.670481 | 0.384837 | 0.397213 | 0.45464 | 0.461411 | 0.694924 |
| GLU151 | 0.846627 | 0.510782 | 0.665978 | 0.495844 | 0.637869 | 0.611063 | 0.639127 | 0.687567 | 0.478611 | 0.397297 | 0.446837 | 0.638713 | 0.921196 | 0.528668 | 0.441785 | 0.605649 | 0.786504 |
| ARG152 | 0.811358 | 0.474561 | 0.738833 | 0.274891 | 0.45777 | 0.646822 | 0.680434 | 0.567712 | 0.583317 | 0.443292 | 0.488436 | 0.491654 | 0.632064 | 0.530455 | 0.420673 | 0.474358 | 0.73718 |
| ARG153 | 0.476261 | 0.584799 | 0.503752 | 0.358927 | 0.548027 | 0.697105 | 0.427453 | 0.481822 | 0.581283 | 0.485202 | 0.38784 | 0.845781 | 0.511994 | 0.423045 | 0.385957 | 0.607277 | 0.692121 |
| GLY154 | 0.457981 | 0.540732 | 0.696816 | 0.681514 | 0.712677 | 0.546832 | 0.603562 | 0.769481 | 0.525337 | 0.396555 | 0.610197 | 0.694529 | 0.481244 | 0.569822 | 0.422928 | 0.865726 | 1.03283 |
| GLU155 | 0.647296 | 0.686637 | 0.559794 | 0.594753 | 0.670061 | 0.568855 | 0.76782 | 0.567225 | 0.58558 | 0.494863 | 0.476638 | 0.919657 | 0.746348 | 0.666004 | 0.622246 | 0.860261 | 0.895821 |
| ASN156 | 0.858597 | 0.652036 | 0.743859 | 0.763567 | 0.588571 | 0.481416 | 0.676233 | 0.593671 | 0.771249 | 0.447181 | 0.495488 | 0.980263 | 0.903536 | 0.796992 | 0.482662 | 0.660878 | 0.711512 |
| PRO157 | 0.866518 | 0.651936 | 0.614133 | 0.902977 | 0.708906 | 0.39028 | 0.899416 | 0.537648 | 0.771856 | 0.613527 | 0.595984 | 1.22105 | 0.617443 | 0.738883 | 0.525103 | 0.581213 | 0.60575 |
| GLY158 | 0.941827 | 0.343251 | 0.628459 | 0.664713 | 0.41495 | 0.52997 | 0.635106 | 0.583001 | 0.621087 | 0.67575 | 0.570056 | 0.779372 | 0.399753 | 0.596271 | 0.569017 | 0.617984 | 0.505087 |
| THR159 | 0.828137 | 0.443127 | 0.535695 | 0.645834 | 0.388554 | 0.609256 | 0.592544 | 0.626732 | 0.559707 | 0.470865 | 0.576103 | 0.728489 | 0.541716 | 0.521412 | 0.56227 | 0.637054 | 0.575984 |
| PRO160 | 0.529163 | 0.407002 | 0.404582 | 0.768503 | 0.473879 | 0.459527 | 0.611503 | 0.424602 | 0.523749 | 0.46095 | 0.540268 | 0.557419 | 0.632501 | 0.421038 | 0.343082 | 0.469416 | 0.381035 |
| ALA161 | 0.466022 | 0.445958 | 0.50873 | 0.48561 | 0.451841 | 0.471625 | 0.57241 | 0.572841 | 0.534249 | 0.613312 | 0.427364 | 0.350331 | 0.572256 | 0.345406 | 0.529213 | 0.465072 | 0.454824 |
| LEU162 | 0.450895 | 0.731114 | 0.505774 | 0.603951 | 0.429601 | 0.467873 | 0.586912 | 0.750686 | 0.589823 | 0.633155 | 0.575732 | 0.509026 | 0.531838 | 0.598436 | 0.800089 | 0.534753 | 0.469776 |
| SER163 | 0.362768 | 0.438939 | 0.462623 | 0.418746 | 0.433708 | 0.458363 | 0.502133 | 0.46412 | 0.427186 | 0.399935 | 0.440285 | 0.431919 | 0.447775 | 0.360186 | 0.465213 | 0.415278 | 0.366885 |
| ILE164 | 0.36879 | 0.41106 | 0.354463 | 0.41595 | 0.415692 | 0.384634 | 0.379654 | 0.465307 | 0.425973 | 0.359978 | 0.3459 | 0.424843 | 0.406253 | 0.407408 | 0.399672 | 0.321286 | 0.392955 |
| GLY165 | 0.327096 | 0.437004 | 0.383093 | 0.478141 | 0.257293 | 0.427581 | 0.328225 | 0.530438 | 0.51424 | 0.382879 | 0.371112 | 0.339871 | 0.480848 | 0.354748 | 0.530141 | 0.378621 | 0.40931 |
| PHE166 | 0.60439 | 0.49167 | 0.771017 | 0.607965 | 0.664649 | 0.613928 | 0.417306 | 0.58117 | 0.577217 | 0.598371 | 0.571089 | 0.418885 | 0.604854 | 0.621611 | 0.854915 | 0.620709 | 0.662487 |
| SER167 | 0.334331 | 0.343685 | 0.326823 | 0.483619 | 0.388202 | 0.448342 | 0.364834 | 0.488108 | 0.521443 | 0.236621 | 0.501354 | 0.439627 | 0.446049 | 0.345317 | 0.475108 | 0.384834 | 0.26716 |
| VAL168 | 0.445916 | 0.34999 | 0.340815 | 0.348208 | 0.334121 | 0.466357 | 0.410647 | 0.416134 | 0.425313 | 0.288747 | 0.374586 | 0.417729 | 0.340103 | 0.375744 | 0.373902 | 0.362263 | 0.318904 |
| ALA169 | 0.367458 | 0.363953 | 0.310336 | 0.380413 | 0.357154 | 0.482472 | 0.355772 | 0.456288 | 0.369267 | 0.273245 | 0.311257 | 0.326607 | 0.388174 | 0.289892 | 0.407512 | 0.323203 | 0.393171 |
| LEU170 | 0.673262 | 0.566524 | 0.420462 | 0.441241 | 0.334716 | 0.6193 | 0.579093 | 0.541999 | 0.47059 | 0.569622 | 0.687998 | 0.387684 | 0.486671 | 0.467766 | 0.738143 | 0.365492 | 0.592149 |
| GLY171 | 0.618739 | 0.326351 | 0.379838 | 0.379065 | 0.341099 | 0.570774 | 0.365809 | 0.37768 | 0.473701 | 0.376006 | 0.310766 | 0.453828 | 0.429758 | 0.427908 | 0.406715 | 0.273366 | 0.517464 |
| HIS172 | 0.608071 | 0.360517 | 0.359273 | 0.366354 | 0.390417 | 0.451694 | 0.3352 | 0.363316 | 0.464765 | 0.399112 | 0.328317 | 0.344633 | 0.403034 | 0.368635 | 0.457949 | 0.261346 | 0.556287 |
| LEU173 | 0.632711 | 0.407167 | 0.425167 | 0.233961 | 0.353288 | 0.686403 | 0.485836 | 0.329566 | 0.402253 | 0.387086 | 0.350601 | 0.514912 | 0.530617 | 0.40227 | 0.606703 | 0.416682 | 0.566512 |
| LEU174 | 0.604091 | 0.461495 | 0.69507 | 0.367585 | 0.58641 | 0.539865 | 0.31821 | 0.525465 | 0.731623 | 0.374057 | 0.338764 | 0.72748 | 0.677952 | 0.412622 | 0.556786 | 0.504374 | 0.373982 |
| GLY175 | 0.466664 | 0.365429 | 0.411683 | 0.307212 | 0.295275 | 0.314578 | 0.260027 | 0.319977 | 0.446671 | 0.307036 | 0.23075 | 0.437395 | 0.429225 | 0.367877 | 0.419826 | 0.301886 | 0.359209 |
| ILE176 | 0.580664 | 0.506841 | 0.533316 | 0.434504 | 0.343144 | 0.436366 | 0.364513 | 0.381402 | 0.425497 | 0.404252 | 0.38037 | 0.367582 | 0.545785 | 0.439613 | 0.530774 | 0.310417 | 0.370398 |
| HIS177 | 0.556969 | 0.527749 | 0.757102 | 0.610486 | 0.424511 | 0.591765 | 0.600217 | 0.528411 | 0.722186 | 0.696118 | 0.701462 | 0.482988 | 0.623838 | 0.683276 | 1.00171 | 0.809543 | 0.533733 |
| TYR178 | 0.731097 | 0.57645 | 0.484427 | 0.42773 | 0.417018 | 0.512216 | 0.362923 | 0.521997 | 0.780463 | 0.470105 | 0.47911 | 0.572357 | 0.636754 | 0.497453 | 0.758723 | 0.761763 | 0.479227 |
| THR179 | 0.699847 | 0.455558 | 0.379801 | 0.421086 | 0.329258 | 0.41299 | 0.343055 | 0.257215 | 0.566348 | 0.342733 | 0.434114 | 0.467837 | 0.347082 | 0.323874 | 0.368035 | 0.54631 | 0.353407 |
| GLY180 | 0.712466 | 0.392373 | 0.40362 | 0.328097 | 0.37487 | 0.382022 | 0.291319 | 0.300174 | 0.388437 | 0.362751 | 0.488537 | 0.366519 | 0.323375 | 0.337183 | 0.334512 | 0.437002 | 0.341092 |
| CYS181 | 0.55723 | 0.350268 | 0.30047 | 0.303954 | 0.368524 | 0.367285 | 0.213923 | 0.29547 | 0.333083 | 0.366314 | 0.364633 | 0.354557 | 0.334867 | 0.299239 | 0.378608 | 0.246298 | 0.361447 |
| SER182 | 0.442287 | 0.310493 | 0.311684 | 0.265886 | 0.364489 | 0.379003 | 0.235353 | 0.260371 | 0.246185 | 0.354425 | 0.271481 | 0.285276 | 0.328301 | 0.243395 | 0.353958 | 0.268139 | 0.371634 |
| MET183 | 0.507127 | 0.344815 | 0.37993 | 0.42662 | 0.354269 | 0.490723 | 0.353393 | 0.439739 | 0.377077 | 0.373887 | 0.272938 | 0.491409 | 0.511174 | 0.229511 | 0.388561 | 0.296301 | 0.457344 |
| ASN184 | 0.360357 | 0.26361 | 0.39341 | 0.407392 | 0.263984 | 0.360497 | 0.272707 | 0.438707 | 0.344428 | 0.292653 | 0.339977 | 0.412435 | 0.46987 | 0.24175 | 0.368725 | 0.370931 | 0.412086 |
| PRO185 | 0.378566 | 0.212749 | 0.469789 | 0.348876 | 0.281928 | 0.428936 | 0.393965 | 0.406568 | 0.274886 | 0.330068 | 0.413184 | 0.423577 | 0.431048 | 0.234506 | 0.311836 | 0.275133 | 0.430125 |
| ALA186 | 0.359011 | 0.249625 | 0.743035 | 0.325516 | 0.334685 | 0.47209 | 0.344444 | 0.455669 | 0.416911 | 0.310386 | 0.565517 | 0.336586 | 0.475082 | 0.43174 | 0.391859 | 0.325397 | 0.451199 |
| ARG187 | 0.336133 | 0.368288 | 0.55393 | 0.296633 | 0.436901 | 0.394131 | 0.310687 | 0.340351 | 0.416315 | 0.399453 | 0.519251 | 0.409394 | 0.390127 | 0.47565 | 0.464022 | 0.423182 | 0.44448 |
| SER188 | 0.269409 | 0.406052 | 0.376031 | 0.290591 | 0.37188 | 0.329471 | 0.439746 | 0.351522 | 0.305457 | 0.284294 | 0.360694 | 0.351551 | 0.349386 | 0.30035 | 0.31995 | 0.404528 | 0.256373 |
| LEU189 | 0.556793 | 0.501203 | 0.416883 | 0.388462 | 0.421212 | 0.433353 | 0.55489 | 0.468653 | 0.364194 | 0.419224 | 0.435253 | 0.445432 | 0.52702 | 0.431369 | 0.314428 | 0.335027 | 0.385692 |
| ALA190 | 0.400336 | 0.272986 | 0.404803 | 0.388915 | 0.317886 | 0.377403 | 0.605042 | 0.370942 | 0.390823 | 0.340175 | 0.299122 | 0.340574 | 0.329853 | 0.430248 | 0.271647 | 0.345954 | 0.357155 |
| PRO191 | 0.454017 | 0.388581 | 0.379406 | 0.371604 | 0.331599 | 0.307914 | 0.532082 | 0.37124 | 0.419993 | 0.29502 | 0.342705 | 0.386393 | 0.27297 | 0.390994 | 0.290654 | 0.36994 | 0.41047 |
| ALA192 | 0.469412 | 0.455631 | 0.307534 | 0.342581 | 0.311241 | 0.34212 | 0.642185 | 0.389394 | 0.392762 | 0.281294 | 0.385633 | 0.410602 | 0.414867 | 0.311304 | 0.256802 | 0.497169 | 0.474079 |
| VAL193 | 0.566043 | 0.536983 | 0.419188 | 0.390755 | 0.331295 | 0.444638 | 0.656664 | 0.455435 | 0.495671 | 0.254659 | 0.449589 | 0.387847 | 0.462302 | 0.427458 | 0.40397 | 0.766553 | 0.551242 |
| VAL194 | 0.501 | 0.402387 | 0.531196 | 0.71376 | 0.36743 | 0.403367 | 0.584589 | 0.489448 | 0.475331 | 0.481408 | 0.615984 | 0.362761 | 0.543358 | 0.464318 | 0.48265 | 0.836302 | 0.688544 |
| THR195 | 0.513737 | 0.506624 | 0.406617 | 0.593741 | 0.372185 | 0.615122 | 0.638976 | 0.474519 | 0.480952 | 0.611935 | 0.526401 | 0.491234 | 0.690716 | 0.52806 | 0.457874 | 0.76543 | 0.600171 |
| GLY196 | 0.508234 | 0.466168 | 0.376274 | 0.428409 | 0.296104 | 0.656593 | 0.690846 | 0.384964 | 0.636963 | 0.669351 | 0.50477 | 0.437906 | 0.642274 | 0.490828 | 0.497718 | 0.579367 | 0.422139 |
| LYS197 | 0.598328 | 0.590382 | 0.473235 | 0.465861 | 0.438293 | 0.902338 | 0.584548 | 0.532695 | 0.514071 | 0.789862 | 0.734448 | 0.530297 | 0.538412 | 0.727055 | 0.64855 | 0.667753 | 0.505016 |
| PHE198 | 0.769997 | 0.472165 | 0.461146 | 0.603378 | 0.478338 | 0.661931 | 0.636955 | 0.429908 | 0.578971 | 0.507209 | 0.612754 | 0.704388 | 0.729788 | 0.604359 | 0.584195 | 0.397588 | 0.494922 |
| ASP199 | 0.705706 | 0.582072 | 0.443586 | 0.510375 | 0.523847 | 0.67618 | 0.550822 | 0.699349 | 0.479244 | 0.524967 | 0.678042 | 0.561689 | 0.836533 | 0.579138 | 0.658038 | 0.473881 | 0.430727 |
| ASP200 | 0.861684 | 0.553338 | 0.76821 | 0.485485 | 0.499992 | 0.505451 | 0.581027 | 0.462337 | 0.509326 | 0.720493 | 0.565337 | 0.703122 | 0.491218 | 0.563602 | 0.687193 | 0.447183 | 0.584515 |
| HIS201 | 0.652015 | 0.477766 | 0.620333 | 0.498357 | 0.39652 | 0.535215 | 0.565062 | 0.444233 | 0.535793 | 0.489367 | 0.715903 | 0.484316 | 0.603791 | 0.535841 | 0.581483 | 0.332653 | 0.485463 |
| TRP202 | 0.722497 | 0.46831 | 0.529931 | 0.359925 | 0.369395 | 0.473235 | 0.4899 | 0.538546 | 0.649722 | 0.557782 | 0.506828 | 0.578496 | 0.463071 | 0.518969 | 0.651692 | 0.437174 | 0.709116 |
| VAL203 | 0.376556 | 0.262053 | 0.344717 | 0.402162 | 0.320914 | 0.427734 | 0.450699 | 0.422357 | 0.428564 | 0.37395 | 0.363894 | 0.400138 | 0.430594 | 0.23954 | 0.362505 | 0.296021 | 0.353421 |
| PHE204 | 0.377842 | 0.450063 | 0.238649 | 0.461003 | 0.331081 | 0.364646 | 0.34564 | 0.353131 | 0.548635 | 0.386627 | 0.415825 | 0.421009 | 0.32121 | 0.450143 | 0.325319 | 0.335825 | 0.335171 |
| TRP205 | 0.441266 | 0.627039 | 0.383591 | 0.590357 | 0.425338 | 0.460064 | 0.425343 | 0.475104 | 0.723274 | 0.678739 | 0.496537 | 0.45991 | 0.564448 | 0.427883 | 0.497307 | 0.35761 | 0.572416 |
| ILE206 | 0.343722 | 0.407476 | 0.355681 | 0.390803 | 0.425494 | 0.412112 | 0.663367 | 0.459395 | 0.685535 | 0.504439 | 0.412028 | 0.475232 | 0.418644 | 0.375549 | 0.423279 | 0.394683 | 0.359118 |
| GLY207 | 0.218644 | 0.331866 | 0.439501 | 0.365964 | 0.302566 | 0.332695 | 0.439112 | 0.335072 | 0.396417 | 0.248729 | 0.395032 | 0.317687 | 0.451576 | 0.292332 | 0.283837 | 0.438149 | 0.307292 |
| PRO208 | 0.289748 | 0.349713 | 0.435721 | 0.350595 | 0.323383 | 0.334287 | 0.438286 | 0.371877 | 0.278463 | 0.321722 | 0.391354 | 0.383511 | 0.419344 | 0.397065 | 0.331246 | 0.402148 | 0.331032 |
| LEU209 | 0.282418 | 0.345395 | 0.460725 | 0.462647 | 0.362456 | 0.420871 | 0.401402 | 0.361092 | 0.353669 | 0.333039 | 0.371773 | 0.408768 | 0.468238 | 0.428884 | 0.475488 | 0.493906 | 0.553017 |
| VAL210 | 0.357573 | 0.237433 | 0.524625 | 0.439361 | 0.407569 | 0.392893 | 0.384007 | 0.463 | 0.447318 | 0.356542 | 0.398709 | 0.427778 | 0.625738 | 0.471778 | 0.378089 | 0.579394 | 0.55777 |
| GLY211 | 0.356445 | 0.316526 | 0.467198 | 0.317939 | 0.211861 | 0.359383 | 0.357182 | 0.44785 | 0.289314 | 0.241643 | 0.445785 | 0.36348 | 0.533304 | 0.462835 | 0.400612 | 0.351995 | 0.496008 |
| ALA212 | 0.390916 | 0.244326 | 0.548538 | 0.316433 | 0.314088 | 0.427603 | 0.464587 | 0.402815 | 0.285 | 0.352759 | 0.567551 | 0.403941 | 0.405403 | 0.867611 | 0.442085 | 0.295993 | 0.469558 |
| ILE213 | 0.383491 | 0.413093 | 0.480947 | 0.420234 | 0.408315 | 0.503558 | 0.407986 | 0.356658 | 0.435039 | 0.584357 | 0.636308 | 0.43261 | 0.380229 | 0.883048 | 0.488455 | 0.522576 | 0.44452 |
| LEU214 | 0.352665 | 0.37298 | 0.656089 | 0.524507 | 0.51936 | 0.407485 | 0.407036 | 0.458692 | 0.437983 | 0.574843 | 0.631076 | 0.535047 | 0.531446 | 0.440787 | 0.578841 | 0.387812 | 0.80126 |
| GLY215 | 0.249374 | 0.443699 | 0.465864 | 0.360474 | 0.414179 | 0.314732 | 0.3512 | 0.351935 | 0.384272 | 0.280211 | 0.608935 | 0.401498 | 0.471944 | 0.327166 | 0.529613 | 0.316381 | 0.495755 |
| SER216 | 0.285135 | 0.48713 | 0.393183 | 0.429117 | 0.432329 | 0.377954 | 0.422698 | 0.424785 | 0.441788 | 0.400552 | 0.411338 | 0.4865 | 0.440368 | 0.40034 | 0.494382 | 0.354278 | 0.397424 |
| LEU217 | 0.42992 | 0.573071 | 0.464649 | 0.436872 | 0.571055 | 0.466272 | 0.509013 | 0.509397 | 0.416069 | 0.606935 | 0.615168 | 0.512996 | 0.440742 | 0.518793 | 0.509451 | 0.465076 | 0.522918 |
| LEU218 | 0.625011 | 0.743204 | 0.601098 | 0.377207 | 0.406134 | 0.499193 | 0.557423 | 0.53218 | 0.458977 | 0.582643 | 0.531465 | 0.471128 | 0.573774 | 0.472176 | 0.497492 | 0.429574 | 0.550158 |
| TYR219 | 0.740681 | 0.610333 | 0.488329 | 0.351317 | 0.352531 | 0.515847 | 0.422315 | 0.47884 | 0.489491 | 0.347269 | 0.487743 | 0.461814 | 0.436626 | 0.391097 | 0.430298 | 0.50357 | 0.528693 |
| ASN220 | 0.729227 | 0.343463 | 0.538706 | 0.463153 | 0.486627 | 0.635541 | 0.824108 | 0.495688 | 0.57049 | 0.439348 | 0.543769 | 0.547108 | 0.507518 | 0.600791 | 0.500642 | 0.466732 | 0.55912 |
| TYR221 | 0.44805 | 0.714294 | 0.543123 | 0.743228 | 0.51642 | 0.621802 | 0.429953 | 0.833632 | 0.726963 | 0.565059 | 0.654717 | 0.625126 | 0.481112 | 0.607875 | 0.793612 | 0.392481 | 0.670364 |
| VAL222 | 0.601635 | 0.591772 | 0.519813 | 0.604544 | 0.364917 | 0.54301 | 0.458733 | 0.599539 | 0.400187 | 0.571963 | 0.558639 | 0.431657 | 0.68026 | 0.368334 | 0.684057 | 0.350651 | 0.738153 |
| LEU223 | 0.689943 | 0.483371 | 0.41589 | 0.411336 | 0.402196 | 0.466601 | 0.496664 | 0.650904 | 0.420545 | 0.599177 | 0.433892 | 0.644395 | 0.737394 | 0.473352 | 0.64751 | 0.4422 | 0.666085 |
| PHE224 | 0.757693 | 0.528052 | 0.617679 | 0.339689 | 0.496958 | 0.618732 | 0.69246 | 0.696653 | 0.436812 | 0.492079 | 0.446194 | 0.57661 | 0.624808 | 0.405822 | 0.462463 | 0.608872 | 0.702941 |
| PRO225 | 0.875899 | 0.492224 | 0.611944 | 0.461719 | 0.555188 | 0.631679 | 0.573305 | 0.69024 | 0.474177 | 0.587467 | 0.770539 | 0.502724 | 0.617248 | 0.400597 | 0.415645 | 0.443126 | 0.683486 |
| PRO226 | 0.873657 | 0.627109 | 0.551075 | 0.447432 | 0.476436 | 0.612364 | 0.623795 | 0.687578 | 0.615964 | 0.550453 | 0.579904 | 0.469716 | 0.536098 | 0.531768 | 0.504615 | 0.385982 | 0.761989 |
| ALA227 | 0.610492 | 0.44388 | 0.382818 | 0.46101 | 0.49086 | 0.622219 | 0.717637 | 0.75118 | 0.512868 | 0.520725 | 0.701575 | 0.48507 | 0.526772 | 0.755945 | 0.531796 | 0.480714 | 0.602702 |
| LYS228 | 0.906503 | 0.575522 | 0.553741 | 0.652163 | 0.898691 | 0.719418 | 0.487655 | 0.969094 | 0.622635 | 0.48731 | 0.804689 | 0.474436 | 0.698562 | 0.618069 | 0.596151 | 0.777196 | 0.592276 |
| SER229 | 0.840493 | 0.683481 | 0.490691 | 0.774339 | 0.721926 | 0.718392 | 0.69808 | 0.802317 | 0.850534 | 0.529312 | 1.07298 | 0.665338 | 0.666971 | 0.476434 | 0.476821 | 0.635586 | 0.467954 |
| LEU230 | 0.862213 | 0.726829 | 0.641435 | 0.815557 | 0.829509 | 0.918388 | 0.853291 | 0.735103 | 1.00926 | 0.909569 | 1.02753 | 0.624258 | 0.766688 | 0.676461 | 1.04229 | 0.946813 | 0.782212 |
| SER231 | 0.862339 | 0.621277 | 0.664545 | 0.540941 | 0.734432 | 0.663716 | 0.930455 | 0.936269 | 1.09246 | 0.816915 | 0.896862 | 0.820194 | 0.842067 | 0.598478 | 1.05446 | 0.606273 | 0.69495 |
| GLU232 | 0.763286 | 0.574728 | 0.564642 | 0.781249 | 0.815016 | 0.624184 | 0.833594 | 0.880039 | 0.907614 | 0.571205 | 0.570107 | 0.572106 | 0.808517 | 0.737668 | 0.637264 | 0.581927 | 0.737988 |
| ARG233 | 0.715225 | 0.721315 | 0.53179 | 0.974001 | 0.800822 | 0.786282 | 0.687292 | 0.651637 | 0.794155 | 0.705911 | 0.499664 | 0.767893 | 0.705985 | 0.626396 | 0.730916 | 0.960471 | 0.660839 |
| LEU234 | 0.632373 | 0.729157 | 0.792208 | 0.647068 | 0.697332 | 0.583191 | 0.648016 | 0.88922 | 0.958179 | 0.684927 | 0.454915 | 0.824656 | 0.697044 | 0.711974 | 0.829886 | 0.677449 | 1.03278 |
| ALA235 | 0.76491 | 0.644262 | 0.809979 | 0.651555 | 0.811948 | 0.523497 | 0.546208 | 1.05201 | 1.04289 | 0.645729 | 0.653258 | 0.813532 | 0.599086 | 0.812956 | 0.932841 | 0.693381 | 0.875437 |
| VAL236 | 0.72356 | 0.675994 | 0.599143 | 0.844554 | 1.01126 | 0.735272 | 0.519007 | 0.95949 | 0.870096 | 0.662245 | 0.592436 | 0.932552 | 0.594915 | 0.890019 | 0.603027 | 0.742063 | 0.772096 |
| LEU237 | 0.632228 | 0.799712 | 0.842885 | 0.722791 | 0.812143 | 0.730596 | 0.76287 | 0.715226 | 0.65547 | 0.760416 | 0.722213 | 0.868212 | 0.559501 | 0.536388 | 0.660331 | 0.739761 | 1.04734 |
| LYS238 | 0.61661 | 0.880416 | 1.12557 | 0.805333 | 1.11345 | 1.03311 | 0.586766 | 0.991922 | 0.806955 | 0.730601 | 0.944898 | 0.782642 | 0.731461 | 0.734981 | 0.74811 | 0.847614 | 1.14023 |
| GLY239 | 0.571618 | 0.923091 | 1.30534 | 1.31697 | 1.29345 | 0.821964 | 0.740793 | 0.907524 | 0.844423 | 0.729286 | 0.8036 | 0.967951 | 0.765442 | 1.08481 | 0.72794 | 0.744342 | 0.966037 |
| LEU240 | 0.510534 | 1.36624 | 1.36575 | 1.06205 | 1.32766 | 0.86066 | 0.904262 | 1.07643 | 1.00093 | 0.87255 | 0.906195 | 0.863627 | 0.923037 | 0.980325 | 0.560898 | 1.46097 | 1.27651 |
